# CHL1 Is a Selective Organizer of the Presynaptic Machinery Chaperoning the SNARE Complex

**DOI:** 10.1371/journal.pone.0012018

**Published:** 2010-08-11

**Authors:** Aksana Andreyeva, Iryna Leshchyns'ka, Michael Knepper, Christian Betzel, Lars Redecke, Vladimir Sytnyk, Melitta Schachner

**Affiliations:** 1 Zentrum für Molekulare Neurobiologie, University of Hamburg, Hamburg, Germany; 2 Institute of Biochemistry and Molecular Biology, University of Hamburg, Hamburg, Germany; 3 School of Biotechnology and Biomolecular Sciences, University of New South Wales, Sydney, New South Wales, Australia; 4 Institute of Neuro- und Sensory Physiology, Heinrich Heine University, Düsseldorf, Germany; 5 Center for Neuroscience, Shantou University Medical College, Shantou, China; 6 Keck Center for Collaborative Neuroscience, Rutgers University, Piscataway, New Jersey, United States of America; Julius-Maximilians-Universität Würzburg, Germany

## Abstract

Proteins constituting the presynaptic machinery of vesicle release undergo substantial conformational changes during the process of exocytosis. While changes in the conformation make proteins vulnerable to aggregation and degradation, little is known about synaptic chaperones which counteract these processes. We show that the cell adhesion molecule CHL1 directly interacts with and regulates the activity of the synaptic chaperones Hsc70, CSP and αSGT. CHL1, Hsc70, CSP and αSGT form predominantly CHL1/Hsc70/αSGT and CHL1/CSP complexes in synapses. Among the various complexes formed by CHL1, Hsc70, CSP and αSGT, SNAP25 and VAMP2 induce chaperone activity only in CHL1/Hsc70/αSGT and CHL1/CSP complexes, respectively, indicating a remarkable selectivity of a presynaptic chaperone activity for proteins of the exocytotic machinery. In mice with genetic ablation of CHL1, chaperone activity in synapses is reduced and the machinery for synaptic vesicle exocytosis and, in particular, the SNARE complex is unable to sustain prolonged synaptic activity. Thus, we reveal a novel role for a cell adhesion molecule in selective activation of the presynaptic chaperone machinery.

## Introduction

Close homologue of L1 (CHL1), a cell adhesion molecule of the immunoglobulin superfamily, regulates migration and differentiation of neurons during ontogenetic development, and enhances neuronal survival [1]–[6]. In mature neurons, CHL1 accumulates in the axonal membrane and regulates clathrin-dependent synaptic vesicle endocytosis [7]. The importance of CHL1 function is underscored by studies showing multiple defects in neurotransmission, long-term potentiation and behavior in CHL1 deficient mice [8]–[12]. In humans, mutations in CHL1 (referred to as CALL) are associated with reduced intelligence, mental retardation and occurrence of schizophrenia [13]–[17]. Prepulse inhibition of the acoustic startle response, a measure of the ability of the central nervous system to gate the flow of sensorimotor information, and working memory, which are reduced in schizophrenic patients, are also reduced in CHL1 constitutively and conditionally deficient mice [8], [10]. It remains unclear, however, how mutations in CHL1 contribute to the development of schizophrenia, the symptoms of which appear only in adulthood, apparently weakening a direct link to CHL1-related abnormalities in ontogenetic brain development and raising the question whether CHL1 may relate more directly to synaptic function.

We have observed a functional link in synaptic vesicle recycling between CHL1 and the 70 kDa heat shock cognate protein (Hsc70) [7], a constitutively expressed chaperone regulating protein folding, transport and sorting [18], [19]. Hsc70 prevents aggregation and degradation of proteins, which transiently acquire vulnerable non-native conformations [20]. In neurons, Hsc70 accumulates in presynaptic boutons and functions as an ATPase that uncoates clathrin from clathrin-coated synaptic vesicles [21].

Since the chaperone activities of Hsc70 are regulated by its co-chaperones we were interested in analyzing the relationship of CHL1 with the co-chaperones of Hsc 70. Among them, the cysteine string protein (CSP), expressed in the brain as αCSP isoform (hereafter denoted CSP), is enriched in synaptic vesicles and regulates neurotransmitter exocytosis [22]–[24]. Another co-chaperone is the small glutamine-rich tetratricopeptide repeat-containing protein (SGT) expressed as ubiquitous (αSGT) and brain specific (βSGT) isoforms. CSP and SGT can directly and simultaneously bind to Hsc70 and upregulate its activity *in vitro* [25]–[26]. Supporting a role of Hsc70, CSP and SGT in protein refolding *in vivo*, CSP deficient mice show progressive degeneration of neuromuscular junctions, Calyx synapses [27] and photoreceptor terminals [28] in early adulthood. We now show that CHL1 associates with presynaptic chaperones in the CHL1/Hsc70/αSGT and CHL1/CSP complexes, the activities of which are directed towards the SNARE complex.

## Results

### CHL1 deficiency results in abnormally reduced chaperone activity in the brain

**Figure 1:**
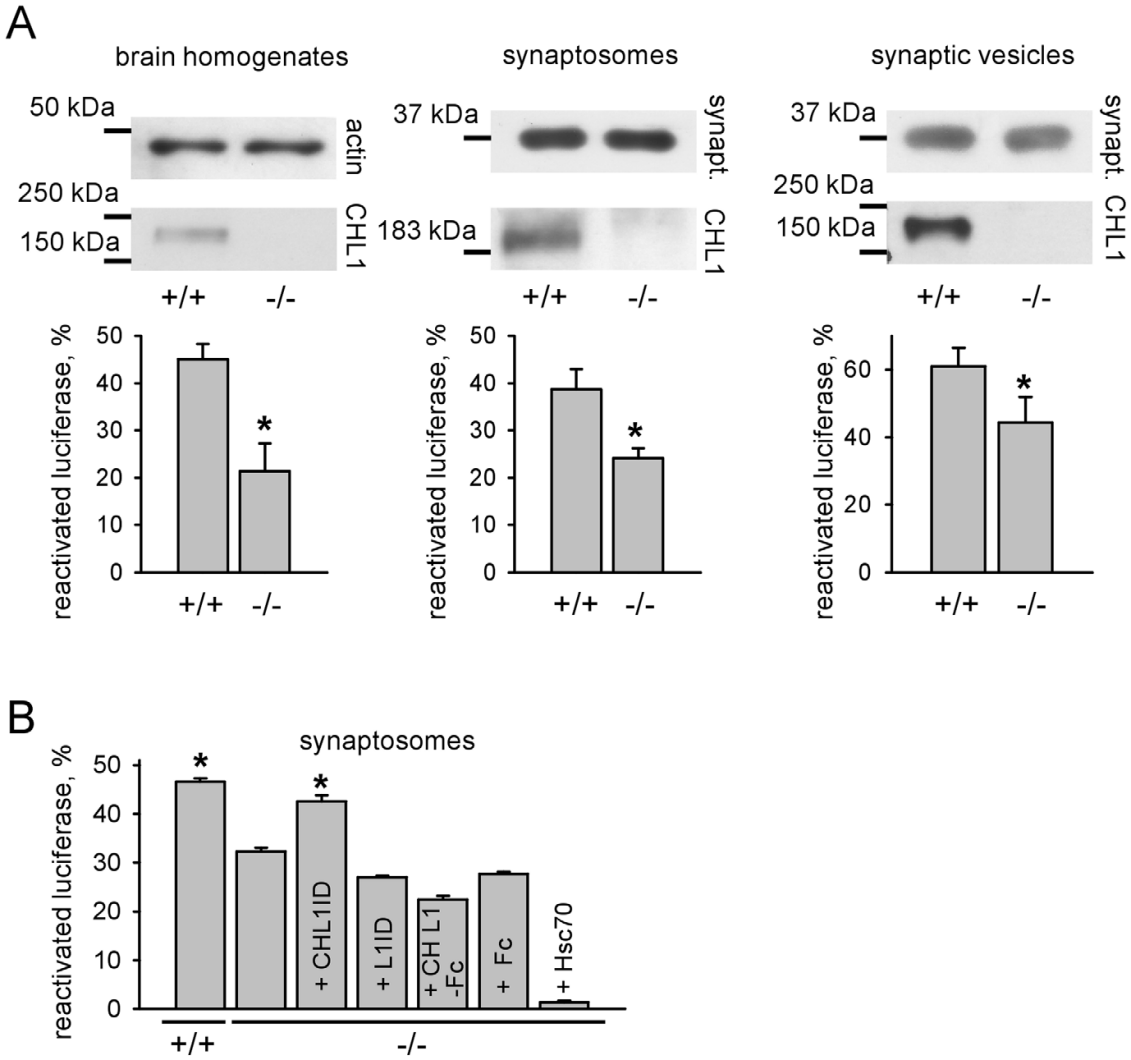
Chaperone activity is reduced in CHL1−/− brains. **A** - Graphs show mean levels of activity ± SEM (n = 6) of luciferase reactivated in CHL1+/+ or CHL1−/− brain homogenates, synaptosomes, or synaptic vesicles normalized to activity levels of native luciferase set to 100%. The probes used for luciferase reactivation assay were probed by Western blot with antibodies against CHL1 and actin (homogenates) or synaptophysin (synaptosomes and synaptic vesicles). Similar levels of actin and synaptophysin in the probes indicate similar total protein levels. Reactivation of luciferase is reduced in CHL1−/− probes. *p<0.05, paired t-test. **B** - Graph shows mean levels of activity ± SEM (n = 6) of luciferase reactivated in CHL1+/+ or CHL1−/− synaptosomes, or in CHL1−/− synaptosomes pre-incubated with recombinant CHL1 intracellular domain (CHL1ID), L1 intracellular domain (L1ID), CHL1 extracellular domain fused to Fc (CHL1-Fc), Fc alone or Hsc70. Values were normalized to activity levels of native luciferase set to 100%. Reactivation of luciferase in CHL1−/− synaptosomes pre-incubated with CHL1ID is increased. *p<0.05, repeated measures ANOVA (Dunnett's multiple comparison test, compared to all other CHL1−/− probes).

Having identified Hsc70 as a binding partner of the intracellular domain of CHL1 [7], we were interested in analyzing whether CHL1 deficiency affects chaperone activity in the adult brain. Chaperone activity was estimated by measuring the efficiency of the reactivation of an artificial substrate, denatured firefly luciferase, in brain homogenates from wild type (CHL1+/+) and CHL1 deficient (CHL1−/−) mice. This method has been used previously to study chaperone activity of Hsc70 and its co-chaperones by measuring bioluminescence of the reactivated luciferase [25], [26]. Luciferase reactivation was reduced in CHL1−/− versus CHL1+/+ brain homogenates (Fig. 1A). Reduced luciferase reactivation in CHL1−/− brains was not due to lower expression of Hsc70, since levels of Hsc70 were upregulated in CHL1−/− brains [7]. Similarly to Hsc70, levels of CSP [7] and αSGT (Fig. S2) were increased in CHL1−/− versus CHL1+/+ brains.

To analyze whether CHL1 deficiency affects chaperone activity in synapses, denatured luciferase was incubated with CHL1+/+ or CHL1−/− synaptosomes or synaptic vesicles. Reactivation of the luciferase was reduced in CHL1−/− versus CHL1+/+ probes (Fig. 1A), indicating that chaperone activity is deficient in CHL1−/− presynaptic boutons. Interestingly, addition of recombinant Hsc70 did not increase but rather inhibited luciferase reactivation in CHL1−/− synaptosomes (Fig. 1B). Reactivation of luciferase activity was increased to CHL1+/+ levels when CHL1−/− synaptosomes were preincubated with the intracellular domain of CHL1 (CHL1ID) (Fig. 1B). In contrast, the intracellular domain of the cell adhesion molecule L1, extracellular domain of CHL1 fused to Fc or Fc alone did not influence luciferase reactivation in CHL1−/− synaptosomes (Fig. 1B). Since CHL1ID has very low intrinsic protein refolding and ATPase activity (Fig. S1), a plausible explanation for this phenomenon is that CHL1ID activates chaperones, probably including Hsc70.

### CHL1 directly interacts with Hsc70, CSP and αSGT

**Figure 2:**
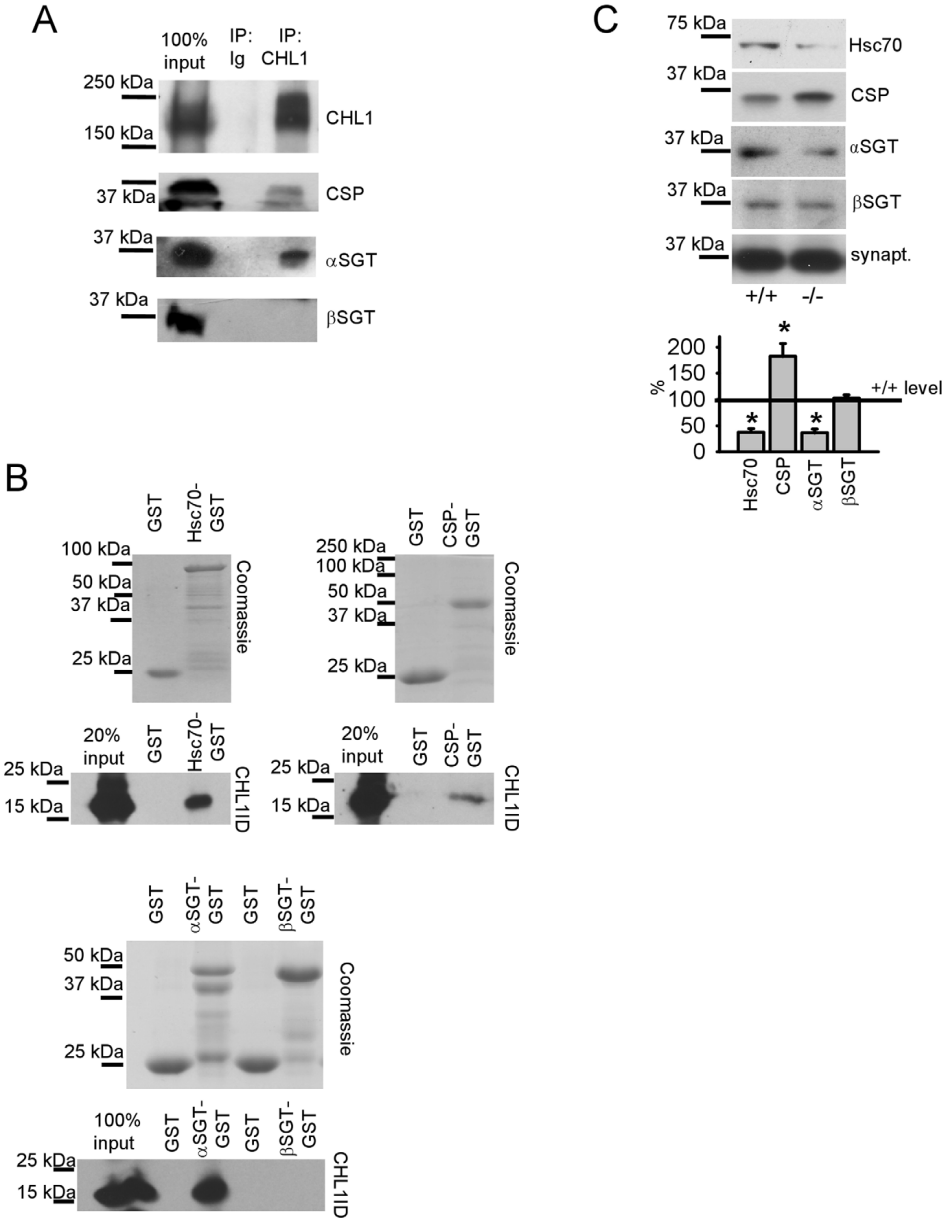
CHL1 interacts with Hsc70, CSP and αSGT. **A** - CHL1 immunoprecipitates (IP) from brain lysates and input material were probed with antibodies against CSP, αSGT and βSGT by Western blot. Mock IP with non-specific immunoglobulins (Ig) was performed for control. CSP and αSGT, but not βSGT co-immunoprecipitated with CHL1. **B** - GST and GST-tagged Hsc70, CSP, αSGT, βSGT immobilized on beads were assayed for their ability to pull down CHL1ID. Coomassie blue stained gels show that levels of Hsc70, CSP, αSGT and βSGT immobilized on beads were similar or lower than levels of GST on control beads. Lower molecular weight products of the recombinant proteins derive from degradation. CHL1ID was pulled down with Hsc70, CSP and αSGT, but not with GST or βSGT. Input shows levels of CHL1ID taken for the pull down assay. **C** - CHL1+/+ and CHL1−/− synaptic vesicles were probed by Western blot with the indicated antibodies. Graphs show mean optical density of the protein bands in CHL1−/− synaptic vesicles ± SEM (n = 6) normalized to the optical density in CHL1+/+ set to 100%. Levels of Hsc70 and αSGT are decreased while levels of CSP are increased in CHL1−/− synaptic vesicles. Levels of βSGT are similar in both genotypes. Synaptophysin (synapt.) served as a loading control. *p<0.05, paired t-test (when compared to CHL1+/+ level (solid line)).

To analyze whether CHL1 interacts with the co-chaperones of Hsc70, co-immunoprecipitation experiments were carried out: 23±2.1 and 46±8.5% of the total pool of CSP and αSGT molecules, respectively, co-immunoprecipitated with CHL1 from brain lysates (Fig. 2A). In contrast, βSGT was not found in the complex with CHL1 (Fig. 2A). Next, we tested whether recombinant CHL1ID could be pulled down with recombinant CSP, αSGT or βSGT, or Hsc70 used as positive control [7]. CHL1ID was pulled down not only with Hsc70 (Fig. 2B), but also with CSP and αSGT, but not with βSGT (Fig. 2B). Thus, CHL1 directly interacts with CSP and αSGT and does not require Hsc70 as a linker.

CHL1/Hsc70 complex formation is important for accumulation of Hsc70 in synaptic vesicles [7]. Similar to Hsc70, levels of αSGT, but not βSGT, were reduced in synaptic vesicles and synaptosomes from CHL1−/− mice (Fig. 2C, Fig. S2) although levels of αSGT were increased in CHL1−/− brain homogenates (Fig. S2). These observations implicate CHL1 in targeting not only Hsc70, but also αSGT to presynaptic boutons and synaptic vesicles. In contrast, CSP levels were increased in CHL1−/− versus CHL1+/+ synaptic vesicles (Fig. 2C), correlating with the overall enhanced expression of CSP in CHL1−/− brain homogenates [7]. CSP, then, appears to be targeted to synaptic vesicles independently of CHL1, probably via palmitoylation of cysteine residues [29].

### CHL1 inhibits formation of the Hsc70/CSP/αSGT complex by promoting formation of the CHL1/CSP and CHL1/Hsc70/αSGT complexes

**Figure 3:**
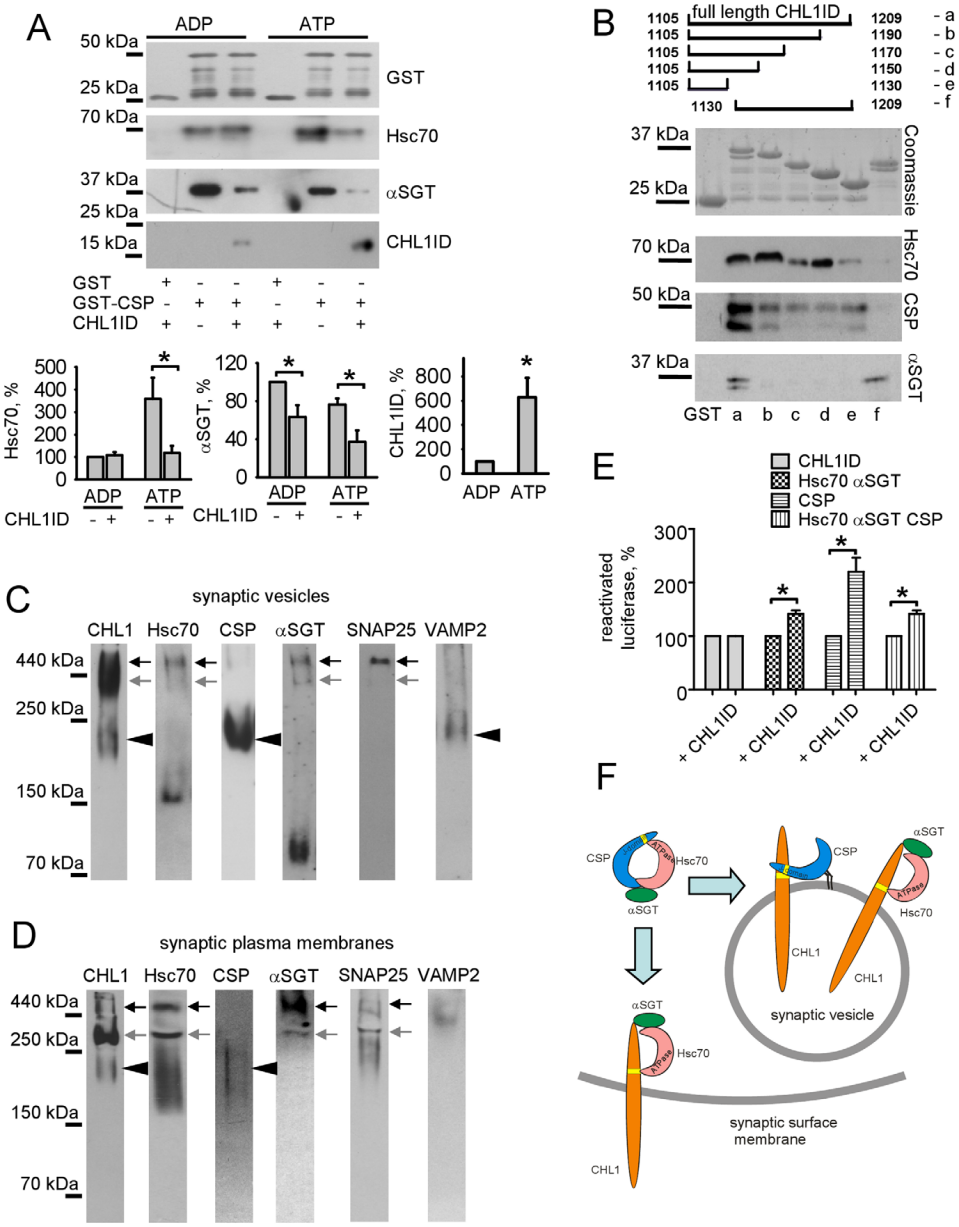
CHL1 recruits Hsc70 and αSGT to synaptic vesicles but inhibits their association with CSP by forming dimeric CHL1/CSP and trimeric CHL1/Hsc70/αSGT complexes. **A** - GST and GST-tagged CSP immobilized on beads were assayed for their ability to pull down Hsc70 and αSGT from a mixture of these proteins in the presence of ADP or ATP with or without CHL1ID. Graphs show mean optical density of the blots ± SEM (n = 6). For quantitation of the binding of Hsc70 and αSGT to CSP (graphs on the left and in the middle, respectively), levels of these proteins bound to CSP in the presence of ADP and absence of CHL1ID were set to 100%. For quantitation of the binding of CHL1ID to CSP (graph on the right), levels of CHL1ID bound to CSP in the presence of ADP were set to 100%. Note that in the presence of ATP, CHL1ID binds to CSP with higher efficiency than in the presence of ADP. CHL1ID inhibits αSGT/CSP complex formation in a nucleotide independent manner and inhibits ATP-dependent formation of the Hsc70/CSP complex. *p<0.05, paired t-test (compared as indicated). **B** - Scheme (top) shows CHL1ID fragments marked alphabetically that were immobilized on beads via the GST tag and assessed for their ability to pull down Hsc70, CSP or αSGT. Coomassie blue stained gel shows that similar levels of CHL1 fragments were immobilized on beads. Lower molecular weight degradation products of the recombinant proteins are also observed. Binding of Hsc70, CSP and αSGT to CHL1ID fragments was analyzed by Western blot with the corresponding antibodies. Note that amino acids 1105–1130 within CHL1ID are necessary to bind Hsc70 and CSP, while amino acids 1190–1209 are necessary to bind αSGT. Double bands recognized by CSP antibodies represent oligomeric forms of recombinant CSP as described previously [51]. **C,D** - Molecular complexes in synaptic vesicles (**C**) and synaptic plasma membranes (**D**) were separated by PAGE under non-denaturing conditions and probed by Western blot with the indicated antibodies. Two or more proteins were considered in a presumed complex, if their bands in the gel overlapped at the apparent molecular weight calculated to be equal or above the total molecular weight of the sum of the weights of the proteins together. In accordance with this criterion, arrows show overlapping bands of CHL1, Hsc70, and αSGT that presumably represent the CHL1/Hsc70/αSGT complex. Note that CHL1/Hc70/αSGT complex migrates at the two levels in the native gel probably due to the multimerization of its components or presence of additional binding partners in the slower migrating level (black arrows) compared to faster migrating level (grey arrows). The higher molecular weight level is more evident in synaptic vesicles, while lower molecular weight level is more evident in synaptic plasma membranes. Arrowheads show overlapping bands of CHL1 and CSP that presumably represent the CHL1/CSP complex. Only complexes of CHL1 together with Hsc70, CSP or αSGT are marked. Note that trimeric Hsc70/CSP/αSGT complexes are not detectable in the gels since CSP bands do not overlap with αSGT bands. In **D**, a CHL1 band overlapping with Hsc70 and CSP immumoreactive bands at ∼230 kDa may contain only the CHL1/CSP complex of ∼230 kDa but not CHL1/Hsc70/CSP or CHL1/Hsc70 complexes with molecular weights above 250 kDa. Note SNAP25 and VAMP2 positive bands, which overlap with CHL1/Hsc70/αSGT and CHL1/CSP complexes, respectively. In synaptic plasma membranes, VAMP2 accumulates in a high molecular weight complex with a slightly different molecular weight than that of the CHL1/Hsc70/αSGT complex. **E** - Graphs show mean levels of activity ± SEM (n = 6) of luciferase reactivated by indicated combinations of chaperones. Values were normalized to luciferase reactivation levels in the absence of the added CHL1ID set to 100%. *p<0.05, paired t-test. **F** - Schematic model diagram of CHL1-containing chaperone complexes. The Hsc70/CSP/αSGT complex dissociates into the CHL1/CSP complex present in synaptic vesicles and the CHL1/Hsc70/αSGT complex present in synaptic vesicles and plasma membranes. HPD tripeptides within the intracellular domain of CHL1 and the J-domain of CSP are marked in yellow.

Since CHL1ID associates with the individual components of the Hsc70/CSP/αSGT complex, we analyzed whether CHL1 associates with or influences formation of the Hsc70/CSP/αSGT complex. Binding of CHL1ID, Hsc70, αSGT to CSP immobilized on beads was analyzed under conditions when CHL1ID, Hsc70 and αSGT were present together in the incubation buffer. Since nucleotides influence binding of CHL1ID to Hsc70 [7] and formation of the trimeric Hsc70/CSP/αSGT complex [25], interactions were analyzed in the presence of ADP or ATP. In the absence of CHL1ID, Hsc70 and αSGT bound to CSP (Fig. 3A). αSGT bound to CSP in a nucleotide-independent manner, while Hsc70 bound to CSP more strongly in the presence of ATP in accordance with a previous report [25]. CHL1ID inhibited binding of αSGT to CSP in a nucleotide-independent manner and totally blocked ATP-dependent binding of Hsc70 to CSP by reducing it to the levels observed in the presence of ADP (Fig. 3A). CHL1ID itself bound to CSP with more prominent binding in the presence of ATP (Fig. 3A). Thus, an inhibitory effect of CHL1ID on Hsc70/CSP/αSGT complex formation is most probably due to binding of CHL1ID to individual components of this complex and masking of sites required for complex assembly. In agreement, CHL1ID and CSP bind to Hsc70 via similar HPD tripeptide based motifs [7], [30], and may compete with each other for binding to Hsc70 (Fig. 3F).

To further analyze the mechanism, by which CHL1 inhibits formation of the Hsc70/CSP/αSGT complex, we attempted to identify binding sites within CHL1ID for Hsc70, CSP, and αSGT. Fragments of CHL1ID that either contained the membrane adjacent portions of CHL1ID of different lengths ranging from 25 to 85 amino acids or the whole CHL1ID without the membrane proximal 25 amino acid stretch, were immobilized on beads and assayed for their ability to pull down Hsc70, CSP or αSGT. All membrane proximal fragments of CHL1ID bound to Hsc70 and CSP, but not to αSGT (Fig. 3B), suggesting that Hsc70 and CSP bind to closely located sequences within the 25 amino acid stretch containing the HPD tripeptide. Thus CSP bound to CHL1ID would sterically interfere with the interaction between CHL1ID and Hsc70 (Fig. 3E). In contrast, deletion of the C-terminal 19 amino acid stretch of CHL1ID abolished the interaction between CHL1ID and αSGT (Fig. 3B), suggesting that this fragment contains the binding site for αSGT. Confirming this finding, the CHL1ID fragment without the membrane adjacent 25 amino acid stretch bound to αSGT, but not to Hsc70 and CSP (Fig. 3B).

To investigate the composition of CHL1-containing protein complexes in synapses we separated protein complexes in synaptic vesicles and synaptic plasma membranes by PAGE under non-denaturing conditions and analyzed them by Western blot. This analysis showed that in synaptic vesicles CHL1 co-migrated with Hsc70, CSP, and αSGT predominantly in trimeric CHL1/Hsc70/αSGT and dimeric CHL1/CSP combinations (Fig. 3C,F). Only low levels of the CHL1/CSP complex were found in synaptic plasma membranes, while this fraction was enriched in the CHL1/Hsc70/αSGT complex (Fig. 3D,F). Tetrameric CHL1/Hsc70/CSP/αSGT or trimeric Hsc70/CSP/αSGT complexes were not detectable in either fraction (Fig. 3C,D).

### CHL1 enhances chaperone activity of Hsc70/αSGT and CSP

To investigate whether CHL1-containing chaperone complexes possess chaperone activity, we studied reactivation of denatured luciferase by Hsc70/αSGT and CSP either in the presence or absence of CHL1ID. Reactivation of luciferase was increased in the presence of CHL1ID (Fig. 3E) suggesting that CHL1ID promotes chaperone activity.

Hsc70/CSP/αSGT has been reported to possess the highest chaperone activity when compared to Hsc70, CSP and αSGT alone [25]. Pre-incubation of this complex with CHL1ID also resulted in increased luciferase reactivation (Fig. 3E). Since CHL1ID induces disassembly of Hsc70/CSP/αSGT into CHL1/Hsc70/αSGT and CHL1/CSP (Fig. 3F), these observations indicate that formation of CHL1/Hsc70/αSGT and CHL1/CSP complexes results in overall higher chaperone activity than that of Hsc70/CSP/αSGT complex.

### CHL1 associates with the SNARE complex proteins

**Figure 4:**
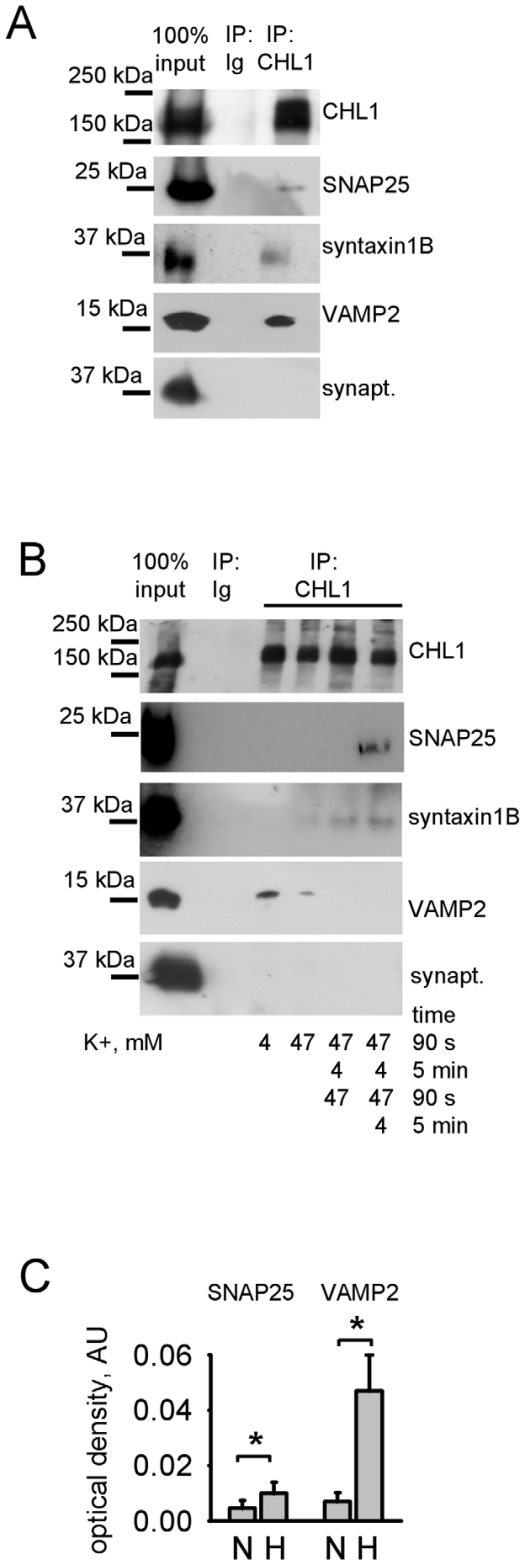
CHL1 interacts with SNARE complex proteins. **A** - CHL1 immunoprecipitates (IP) from brain lysates and input material were probed with the indicated antibodies by Western blot. Mock IP with non-specific rabbit immunoglobulins (Ig) was performed for control. SNAP25, syntaxin1B, and VAMP2, but not synaptophysin co-immunoprecipitated with CHL1. **B** - CHL1 was immunoprecipitated (IP) from synaptosomes that were mock-stimulated with 4 mM K+, or stimulated one or two times with 47 mM K+ for 90 s with or without 5 min recovery in 4 mM K+ after stimulations (as indicated). CHL1 immunoprecipitates and input material were then probed by Western blot with indicated antibodies. Mock IP with non-specific immunoglobulins (Ig) was performed for control. Note that no co-immunoprecipitation of SNAP25 and syntaxin 1B with CHL1 is observed in mock-stimulated synaptosomes even after high exposure of the blots. Stimulation induces co-immunoprecipitation of SNAP25 and syntaxin1B, and decreases levels of co-immunoprecipitated VAMP2. **C** - CHL1ID (1.9 µM) was immobilized on plastic and assayed by ELISA for the ability to bind SNAP25 (4 µM) and VAMP2 (6 µM) either in native form (N) or after exposure to 42°C (H). CHL1ID binds stronger to non-native SNAP25 and VAMP2. *p<0.05, paired t-test (n = 6).

Further analysis showed that the CHL1/Hsc70/αSGT-containing band observed under non-denaturing conditions overlapped with the SNAP25-immunoreactive band in synaptic vesicles and synaptic plasma membranes (Fig. 3C,D). In contrast, the CHL1/CSP-containing band overlapped with the VAMP2-immunoreactive band in synaptic vesicles (Fig. 3C), but not in synaptic plasma membranes (Fig. 3D). In agreement, analysis of CHL1 immunoprecipitates from brain lysates by Western blot with antibodies against the SNARE complex proteins showed that 17±6.8%, 15±3.1%, 22±11.9% of the total pool of the SNARE complex proteins SNAP25, syntaxin1B, and VAMP2, respectively, co-immunoprecipitated with CHL1 (Fig. 4A). Another synaptic vesicle associated protein, synaptophysin, did not co-immunoprecipitate with CHL1 (Fig. 4A).

The interaction between CHL1 and SNAP25 or syntaxin1B, however, was not detectable in synaptosomes isolated from brain tissue (Fig. 4B) suggesting that the CHL1/SNAP25 and CHL1/syntaxin1B complexes detectable in brain homogenates are from extrasynaptic compartments. Since SNARE proteins redistribute to extrasynaptic sites following synapse activation [31], we analyzed whether interactions between CHL1 and the SNARE complex proteins depend on synaptic activity by treating synaptosomes for 90 s with a buffer containing 47 mM K+ to depolarize synaptic plasma membranes and induce synaptic vesicle exo- and endocytosis. Western blot analysis of CHL1 immunoprecipitates from stimulated synaptosomes showed that SNAP25 and syntaxin1B co-immunoprecipitated with CHL1 from synaptosomes activated with 47 mM K+ (Fig. 4B). The extent of this co-immunoprecipitation was increased in synaptosomes repetitively stimulated with 47 mM K+ with short intervals of recovery in 4 mM K+ (Fig. 4B), suggesting that synaptic vesicle recycling triggers formation of the CHL1/SNAP25 and CHL1/syntaxin1B complexes. In contrast to SNAP25 and syntaxin1B, the association of CHL1 with VAMP2 was strongest in non-stimulated synaptosomes and diminished following stimulation with 47 mM K+ (Fig. 4B).

Interestingly, CHL1ID showed low binding to purified SNAP25 or VAMP2 immobilized on plastic (Fig. 4C). Binding of CHL1ID was enhanced, however, when SNAP25 and VAMP2 were exposed to 42°C (Fig. 4C) to induce non-native conformations (see below). Thus, the affinity of CHL1ID to SNARE proteins appears to depend on their conformation, and acquisition of non-native conformations by SNARE proteins during synaptic vesicle recycling may account for the increased association of CHL1ID with these proteins in stimulated versus non-stimulated synaptosomes. The fact that CHL1ID interacts preferentially with non-native SNARE proteins may also account for relatively low co-immunoprecipitation efficiency of these proteins with CHL1.

### CHL1/CSP and CHL1/Hsc70/αSGT complexes chaperone SNARE complex components

To obtain insights into the role of the interactions between CHL1 and the SNARE complex proteins, we analyzed whether SNARE complex proteins can activate CHL1-containing chaperone complexes under conditions close to physiological. Recombinant SNAP25 and VAMP2 were exposed to 42°C for 10 min in the presence of high concentrations of bivalent ions which can be found in highly active synaptic boutons. We have deliberately chosen conditions, which are close to those in diseased or highly stressed organisms, since mutations in CHL1 are associated with stress related disorders such as schizophrenia. Since ATPase activity of chaperones is directly linked to chaperoning of the substrate proteins and thus can serve as a marker of the chaperone activity, ATPase activity of CHL1-containing complexes was analyzed by estimating levels of inorganic phosphate released from ATP by these complexes in the presence of heat-treated SNAP25 and VAMP2. ATPase activity was always compared to the basal ATPase activity in the presence of native SNAP25 and VAMP2, which had not been exposed to heat.

**Figure 5:**
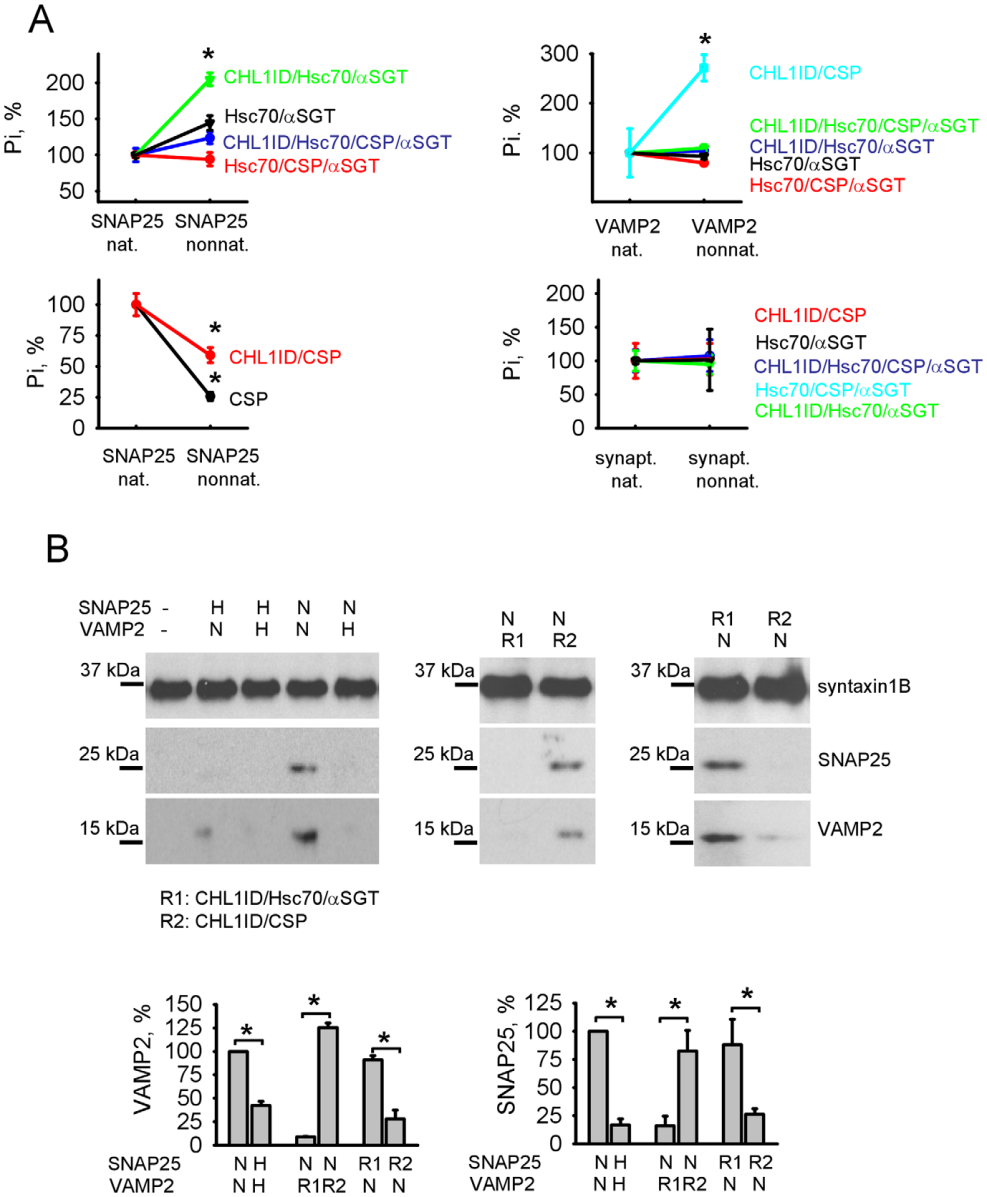
SNARE complex proteins selectively activate the chaperone activity of CHL1-containing chaperone assemblies. **A** - Graphs show mean levels ± SEM of free inorganic phosphate (Pi) released from ATP (ATPase activity) by the indicated combinations of Hsc70, CSP, αSGT, and CHL1ID in response to native or heat-treated non-native SNAP25, VAMP2, or synaptophysin. The complete list of all tested combinations of CHL1ID, Hsc70, CSP, and αSGT is shown in Table S1. Note that non-native SNAP25 and VAMP2 activate ATPase activity of CHL1ID/Hsc70/αSGT and CHL1ID/CSP complexes, respectively. *p<0.05, paired t-test (compared to the ATPase activity in the presence of native proteins set to 100% (n≥6)). **B** - Syntaxin1B was immunoprecipitated from synaptosomes and assayed for the ability to pull down heat-treated non-native (H) or native (N) SNAP25 and VAMP2. In indicated cases, non-native proteins were preincubated with CHL1ID/Hsc70/αSGT (R1) or CHL1ID/CSP (R2) complexes. Graphs show quantitation of the binding of SNAP25 and VAMP2 to syntaxin1B ± SEM (n = 6) with the binding efficiency for both proteins in the native conformation set to 100%. Native SNAP25 and VAMP2 form a complex with syntaxin1B, while exposure to heat of at least one component inhibits the pull down efficiency. Preincubation of non-native SNAP25 with CHL1ID/Hsc70/αSGT and non-native VAMP2 with CHL1ID/CSP complexes increases the efficiency of the SNARE complex assembly. *p<0.05, paired t-test (compared as indicated).

Analysis of ATPase activity of CHL1ID, Hsc70, CSP and αSGT alone or various combinations of these proteins showed that heat-treated VAMP2 specifically increased ATPase activity of only the CHL1ID/CSP complex, with ATPase activity of this complex being ∼3 times higher in the presence of heat-treated versus native VAMP2 (Fig. 5A). These observations are in agreement with previous reports showing that CSP possesses Hsc70-independent chaperone activity both *in vitro* and *in vivo* [30], [32]. In contrast, heat-treated SNAP25 activated only the CHL1ID/Hsc70/αSGT complex, with ATPase activity of this complex being ∼2 times higher in the presence of heat-treated versus native SNAP25 (Fig. 5A). A slight, but significant increase in ATPase activity in the presence of heat-treated SNAP25 was also observed for the Hsc70/αSGT combination (Fig. 5A). These data indicate that SNAP25 and VAMP2 are not only able to acquire non-native chaperone-activating conformations, but also suggest a high specificity in their interactions with the chaperones.

Interestingly, non-native SNAP25 inhibited the basal ATPase activity of CSP alone or in combination with CHL1ID (Fig. 5A). A plausible explanation for this finding is that SNAP25 inhibits the chaperone activity of CSP during synaptic vesicle exocytosis, when surface plasma membrane enriched SNAP25, which unfolds during fusion of synaptic vesicle with surface membranes, is in the vicinity of synaptic vesicle localized CSP. This inhibition may then be required to allow VAMP2 to change its conformation for synaptic vesicle exocytosis. None of the chaperone complexes was activated in the presence of heat-treated synaptophysin (Fig. 5A).

Next we assessed binding of recombinant SNAP25 and VAMP2 to synatxin1B immunopurified from mouse brains. Non-treated SNAP25 and VAMP2 bound syntaxin1B immobilized on beads (Fig. 5B). When either SNAP25 or VAMP2 were pre-exposed to 42°C for 10 min, binding of both proteins was strongly reduced when compared to binding of non-treated proteins (Fig. 5B). Binding of non-treated SNAP25/heat-treated VAMP2 to synatxin1B was restored when heat-treated VAMP2 was pre-incubated with the CHL1ID/CSP complex, but not with the CHL1ID/Hsc70/αSGT complex (Fig. 5B). In contrast, binding of heat-treated SNAP25/non-treated VAMP2 was restored when heat-treated SNAP25 was pre-incubated with the CHL1ID/Hsc70/αSGT complex, but not with the CHL1ID/CSP complex (Fig. 5B).

Combined observations indicate that VAMP2 and SNAP25 are substrates for two distinct chaperone complexes, CHL1/CSP and CHL1/Hsc70/αSGT, respectively, suggesting that these chaperones could assist VAMP2 and SNAP25 in assembly of the SNARE complex.

### CHL1ID/Hsc70/αSGT and CHL1ID/CSP chaperone complexes dissolve aggregates formed by non-native SNAP25 and VAMP2

**Figure 6:**
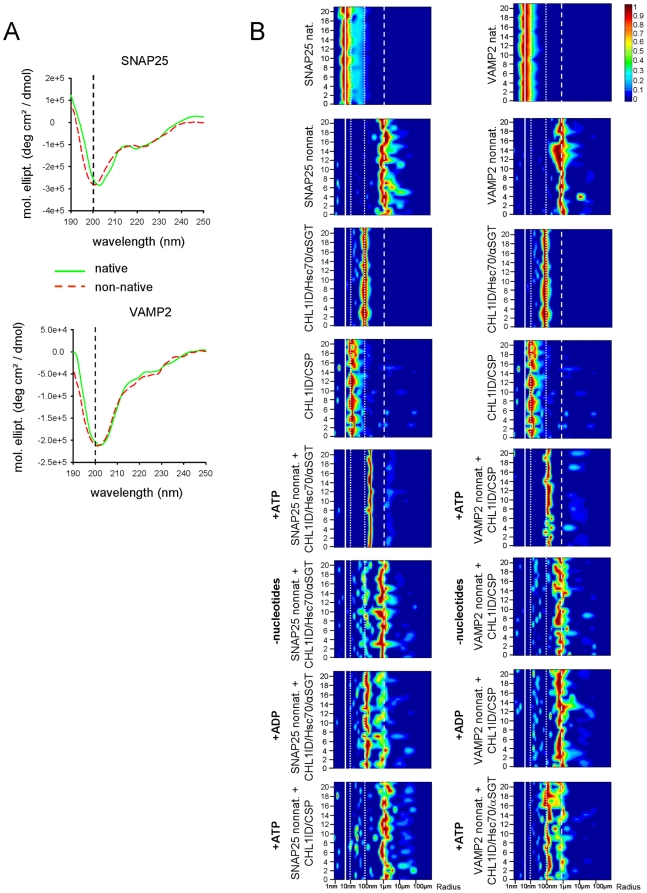
CHL1ID/Hsc70/αSGT and CHL1ID/CSP chaperone complexes dissolve aggregates of SNAP25 and VAMP2, respectively. **A** – CD spectra of native and heat-treated non-native SNAP25 and VAMP2. Exposure of the proteins to 42°C for 10 min did not affect the secondary structure of the proteins, as revealed by almost identical CD spectra. A minor shift of the characteristic minimum towards 200 nm might indicate a slight increase in their random coil structure content. **B** - Distribution of the radii of the protein complexes as measured by DLS. Data from 22 consecutive DLS measurements are shown with signal intensities displayed in pseudocolor. Heat exposure results in aggregation of SNAP25 and VAMP2 (solid and dashed lines mark the size of particles formed by proteins in native and non-native conformations, respectively). CHL1ID, Hsc70 and αSGT form a complex, which is larger than the complex formed by CHL1ID and CSP. The sizes of these complexes are marked by dotted lines (the same data are shown in two columns for reference). Note, that SNAP25 and VAMP2 aggregates are dispersed in the presence of ATP by CHL1ID/Hsc70/αSGT and CHL1ID/CSP, respectively. Protein aggregates are not dispersed in the absence of nucleotides or in the presence of ADP. Under these conditions, the aggregates and CHL1ID/Hsc70/αSGT complex are seen as assemblies of two distinct sizes, with the smaller CHL1ID/CSP complex being obscured by the larger aggregates, because the scattering intensity is proportional to the particle radius in the power of 3 [52]. SNAP25 aggregates are not dispersed by CHL1ID/CSP, and VAMP2 aggregates are not dispersed by CHL1ID/Hsc70/αSGT.

To gain insights into the functions of the CHL1-containing chaperones, we analyzed changes in the structures of SNAP25 and VAMP2 following thermal exposure. Circular dichroism (CD) spectroscopy showed that SNAP25 and VAMP2 are proteins largely composed of random coil structure (Fig. 6A) as observed previously [33], [34]. In solution, both proteins are characterized by hydrodynamic radii of 5.2±0.5 nm (SNAP25) and 5.6±0.3 nm (VAMP2), as revealed by dynamic light scattering (DLS) measurements (Fig. 6B). For monomeric SNAP25 (M_w_ = 23315 Da) and VAMP2 (M_w_ = 12690 Da), hydrodynamic radii of 2.15 nm and 1.76 nm, respectively, are expected, assuming a roughly globular shape [35]. However, the undefined random coil structure of both proteins strongly decreases the diffusion of the molecules in solution, resulting in a significantly increased apparent hydrodynamic radius. On the other hand, the formation of small oligomeric states can not be excluded. When SNAP25 and VAMP2 were exposed to 42°C for 10 min, the secondary structure of the proteins was not affected significantly, as revealed by almost identical CD spectra. Only marginal shifts of the predominant minima towards 200 nm with respect to the spectra for native proteins have been observed that could indicate minor changes in the protein conformation (Fig. 6A). Remarkably, exposure to 42°C resulted in a pronounced aggregation of SNAP25 and VAMP2 reflected by formation of large assemblies with hydrodynamic radii of ∼1 µm (1134.30±191.47 nm for SNAP25 and 904.12±142.90 nm for VAMP2) as measured by DLS (Fig. 6B). This observation suggested that heat treatment induced acquisition of non-native conformations by both SNAP25 and VAMP2, which cannot be detected by CD spectroscopy. When non-native SNAP25 was incubated with the CHL1ID/Hsc70/αSGT complex in the presence of ATP, these large assemblies were partially dispersed, resulting in particles with a radius of 171.05±15.87 nm, probably representing complexes of SNAP25 and chaperones (Fig. 6B). In contrast, assemblies of non-native SNAP25 were not dispersed by the CHL1ID/Hsc70/αSGT complex in the absence of nucleotides or in the presence of ADP, or by the CHL1ID/CSP complex in the presence of ATP. Similarly, when non-native VAMP2 was incubated with the CHL1ID/CSP complex in the presence of ATP, VAMP2 aggregates were partially dispersed forming assemblies with a radius of 150.00±30.41 nm (Fig. 6B). In contrast, aggregates of non-native VAMP2 were not dispersed by the CHL1ID/CSP complex in the absence of nucleotides or in the presence of ADP, or by the CHL1ID/Hsc70/αSGT complex in the presence of ATP.

The combined data indicate that CHL1ID/Hsc70/αSGT and CHL1ID/CSP complexes specifically disperse aggregates formed by non-native SNAP25 and VAMP2, respectively.

### CHL1 deficiency results in increased degradation of the SNARE complex proteins in response to stress

**Figure 7:**
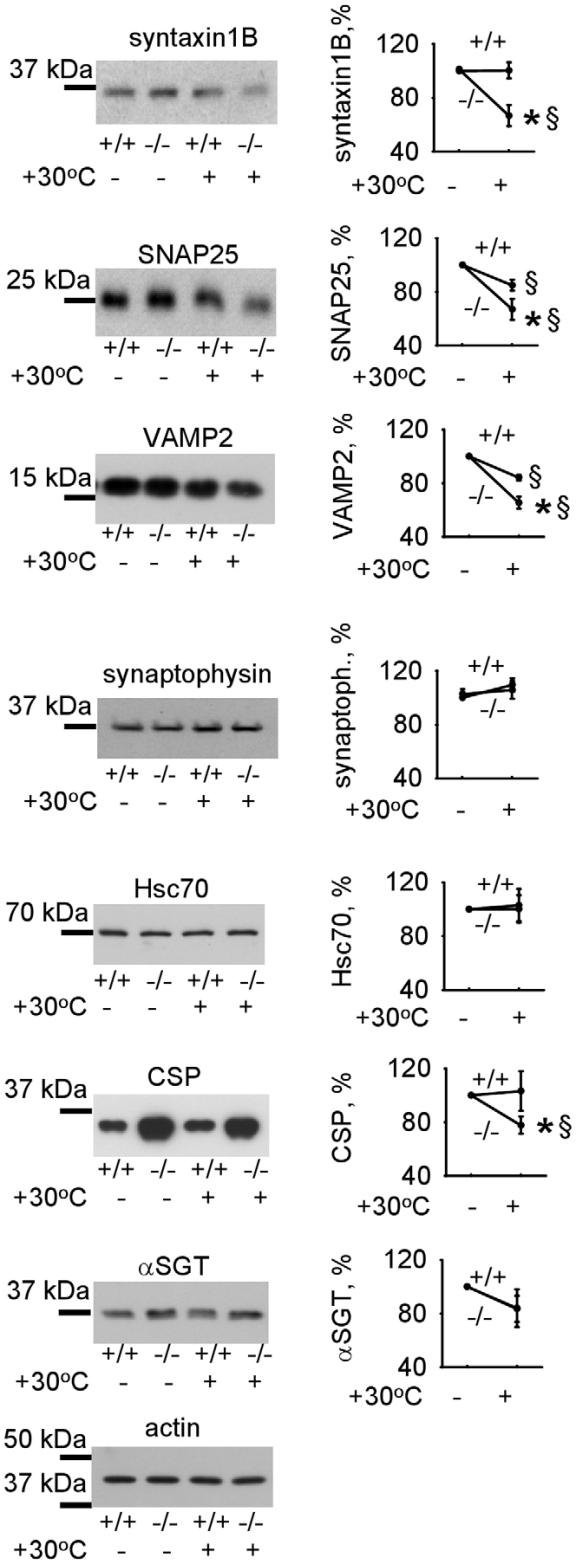
CHL1 deficiency results in the increased degradation of the SNARE complex proteins under elevated temperatures. CHL1+/+ and CHL1−/− brain homogenates either freshly prepared or pre-incubated at 30°C for 30 minutes were probed by Western blot with the indicated antibodies. Note a more pronounced decline in the levels of syntaxin1B, SNAP25 and VAMP2, but not synaptophysin in heat-exposed CHL1−/− versus CHL1+/+ brain homogenates. Levels of Hsc70, CSP and αSGT are similar or higher in CHL1−/− versus CHL1+/+ brain homogenates exposed to 30°C. Actin served as a loading control. Graphs show mean optical density of the blots ± SEM (n = 6) with optical density in non-treated homogenates set to 100%. *, § p<0.05, paired t-test (CHL1−/− versus CHL1+/+ heat-treated brain homogenates (*), and non-treated versus treated groups of the same genotype (§) were compared).

Incorrect folding and aggregation result in protein degradation by cellular proteases [36], [37]. Thus, an analysis of protein degradation rates can provide an estimation of the efficiency of the chaperones. Exposure to 30°C, i.e. a temperature which is even lower than the one used in the *in vitro* assay, induced a pronounced degradation of SNARE complex proteins in CHL1−/− brain homogenates, while only mildly affecting levels of these proteins in CHL1+/+ brain homogenates (Fig. 7).

**Figure 8:**
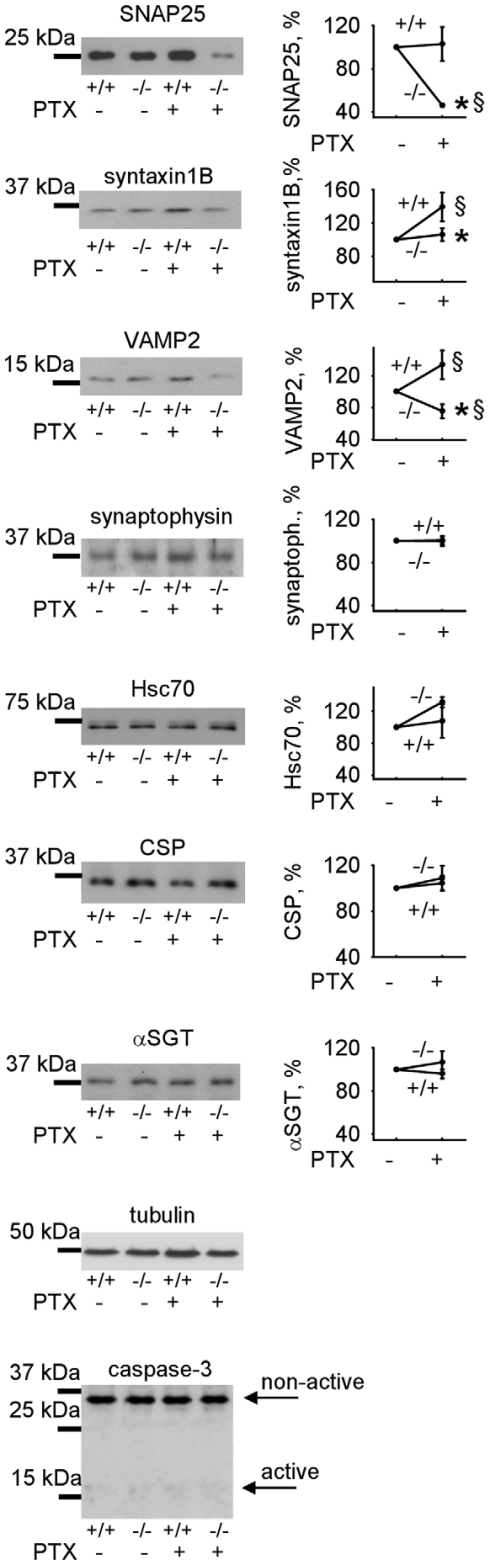
Levels of SNARE proteins are reduced in CHL1−/− versus CHL1+/+ neurons subjected to high levels of synaptic activity. CHL1+/+ and CHL1−/− cultured hippocampal neurons were stimulated with 50 µM PTX applied for 30 min. Lysates of non-treated and PTX treated neurons were probed by Western blot with the indicated antibodies. Graphs show mean optical density of the blots ± SEM (n = 6) with signals in non-treated neurons set to 100%. Note reduced levels of SNAP25, syntaxin1B, and VAMP2, but not synaptophysin in stimulated CHL1−/− versus stimulated CHL1+/+ neurons. PTX application did not induce apoptosis as indicated by unchanged levels of non-active and active caspase-3 in both genotypes. Tubulin served as a loading control. *,§ p<0.05, paired t-test (PTX stimulated CHL1−/− versus CHL1+/+ neurons (*), and PTX stimulated versus non-stimulated neurons of the same genotype (§) were compared).

We next analyzed whether synaptic activity influences the levels of the SNARE complex proteins. CHL1+/+ and CHL1−/− cultured hippocampal neurons were treated for 30 min with 50 µM picrotoxin (PTX), an inhibitor of GABA_A_ receptors that induces seizure-like activity in neurons. Western blot analysis of cell lysates of these cultures showed that levels of SNAP25 and VAMP2, but not synaptophysin were strongly reduced in PTX-treated versus non-treated CHL1−/− neurons (Fig. 8). PTX stimulation did not affect the SNARE complex protein levels in CHL1+/+ neurons (Fig. 8). However, levels of VAMP2 and syntaxin1B were increased in stimulated CHL1+/+ neurons, probably due to enhanced synthesis of these proteins. Although levels of syntaxin1B were not changed in PTX-treated versus non-treated CHL1−/− neurons, they were lower than the levels of syntaxin1B in PTX-treated CHL1+/+ neurons (Fig. 8). These results again suggest higher degradation of syntaxin1B in activated CHL1−/− neurons when compared to CHL1+/+ neurons. We cannot exclude, however, that synthesis of the SNAP25, syntaxin 1B and VAMP2 in response to PTX stimulation is also affected by CHL1 deficiency. PTX treatment was not associated with enhanced apoptosis in CHL1−/− neurons as indicated by unchanged levels of active caspase-3 in stimulated versus non-stimulated neurons of both genotypes (Fig. 8). Levels of Hsc70, CSP and αSGT were also not affected by PTX stimulation in both genotypes (Fig. 8). Thus, our data indicate that CHL1 deficiency results in enhanced susceptibility of the synaptic vesicle fusion machinery to stressful conditions, such as high synaptic activity.

### Aggregation and degradation of the SNARE complex proteins are increased in CHL1−/− mice

**Figure 9:**
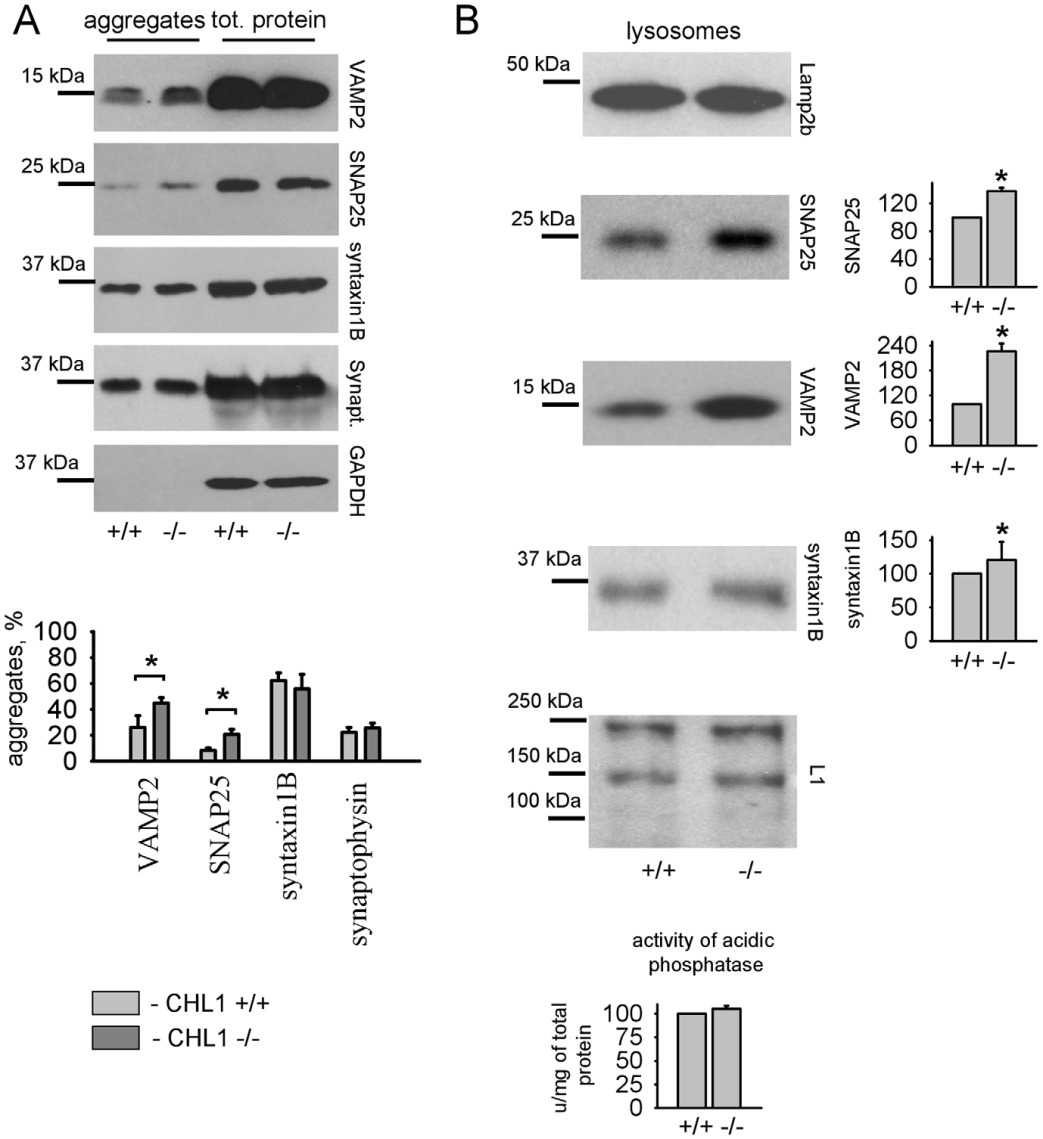
CHL1 deficiency results in aggregation and lysosome targeting of VAMP2 and SNAP25. **A** - Protein aggregates and total protein from CHL1+/+ and CHL1−/− brains were probed by Western blot with the indicated antibodies. Note that higher levels of VAMP2 and SNAP25 are present in aggregates from CHL1−/− brain homogenates. Graph shows mean protein levels in aggregates ± SEM (n = 6) normalized to the total protein level. *p<0.05, paired t-test. **B** - CHL1+/+ and CHL1−/− lysosomes were probed by Western blot with antibodies against the lysosome associated proteins Lamp2b, SNAP25, VAMP2 and syntaxin1B. L1 served as a loading control. Graphs on the right show mean levels ± SEM of SNAP25, VAMP2 and syntaxin1B in lysosomes with levels in CHL1+/+ lysosomes set to 100%. Note similar levels of Lamp2b in CHL1+/+ and CHL1−/− lysosomes indicating similar efficiency of lysosome isolation. Graph (below) shows mean levels ± SEM of acidic phosphatase activity in CHL1+/+ and CHL1−/− lysosomes that was similar in lysosomes of both genotypes, again indicating that similar levels of lysosomes were isolated. Levels of SNAP25, VAMP2, syntaxin1B, but not L1 are increased in CHL1−/− lysosomes. *p<0.05, paired t-test.

We next analyzed whether abnormal SNARE protein aggregation can be detected in CHL1−/− mice. Protein aggregates were isolated from the total protein pool of CHL1+/+ and CHL1−/− brains by means of centrifugation. Western blot analysis showed that ∼20% and 10% of VAMP2 and SNAP25, respectively, were present as aggregates in CHL1+/+ brain homogenates (Fig. 9A). The levels of aggregated VAMP2 and SNAP25 were ∼2 fold higher in CHL1−/− brain homogenates (Fig. 9A). The levels of aggregated syntaxin 1B, synaptophysin were similar in either genotype (Fig. 9A). No detectable aggregates of GAPDH were found in either genotype (Fig. 9A).

The levels of SNAP25 and VAMP2 were also increased in lysosomes isolated from CHL1−/− brains (Fig. 9B), suggesting increased targeting for degradation. Syntaxin 1B levels were only slightly increased in CHL1−/− lysosomes, while levels of the cell adhesion molecule L1, which served as a loading control, were similar in both genotypes (Fig. 9B).

### CHL1 deficiency is associated with reduced reassembly of the SNARE complex components and inhibition of synaptic vesicle recycling following prolonged and stressful synapse activity

**Figure 10:**
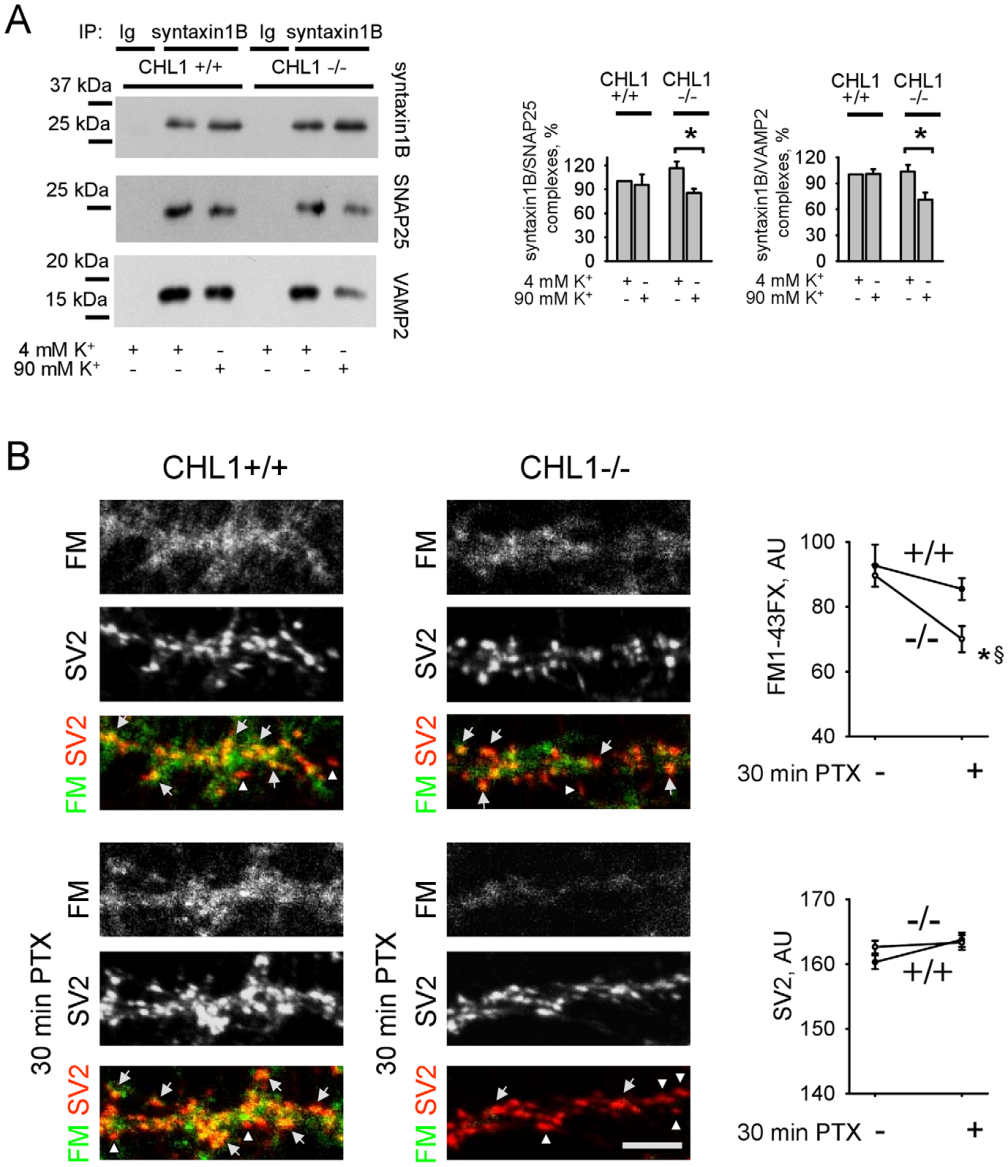
Sustainability of the synaptic vesicle recycling machinery is reduced in CHL1−/− neurons. **A** - Syntaxin1B immunoprecipitates (IP) from CHL1+/+ and CHL1−/− synaptosomes, treated with 4 mM or 90 mM K+, were probed by Western blot with antibodies against synataxin1B, SNAP25 and VAMP2. Graphs show mean optical densities of the blots ± SEM (n = 6) with the levels of SNAP25 and VAMP2 that co-immunoprecipitate with syntaxin1B from 4 mM K+ treated CHL1+/+ synaptosomes set to 100%. Exposure to 90 mM K+ reduces levels of SNAP25 and VAMP2 that co-immunoprecipitate with syntaxin1B from CHL1−/− synaptosomes. *p<0.05, paired t-test (compared as indicated). **B** - CHL1+/+ and CHL1−/− cultured hippocampal neurons that were either non-treated or pre-incubated with 50 µM PTX for 30 min were then incubated with PTX for 10 min in the presence of FM1-43FX. After fixation, neurons were co-labeled with antibodies against SV2. Note FM1-43FX loaded (yellow on overlay images, arrows) and non-loaded (red on overlay images, arrowheads) SV2 positive synaptic boutons. Note, that FM1-43FX non-loaded synapses are present in all groups. However, number of FM1-43FX non-loaded synapses is increased and levels of FM1-43FX in FM1-43FX loaded synapses are reduced in CHL1−/− versus CHL1+/+ neurons pre-treated with PTX. Graphs show mean FM1-43FX and SV2 fluorescence levels ± SEM in SV2 accumulations (N>30 images of neurons with n>200 SV2 positive synaptic boutons per image were analyzed in each group). *,§ p<0.05, t-test (PTX stimulated CHL1−/− versus CHL1+/+ neurons (*), and PTX stimulated versus non-stimulated neurons of the same genotype (§) were compared).

To investigate whether the ability to reassemble the SNARE complex is affected by CHL1 deficiency, we analyzed co-immunoprecipitations of SNAP25 and VAMP2 with syntaxin1B from synaptosomes. SNAP25 and VAMP2 co-immunoprecipitated with syntaxin1B with a similar or slightly higher efficiency from non-stimulated CHL1−/− versus CHL1+/+ synaptosomes (Fig. 10A). However, following stimulation of synaptosomes for 90 s with 90 mM K+ to induce synaptic vesicle exo- and endocytosis, the ability of VAMP2 and SNAP25 to associate with syntaxin1B was reduced in CHL1−/− synaptosomes by ∼25% compared to non-stimulated CHL1−/− synaptosomes (Fig. 10A). These data indicate that CHL1 deficiency results not only in enhanced degradation of SNARE complex proteins, but also in reduced ability of these proteins to re-associate in the SNARE complex following synaptic vesicle exocytosis.

To assess the impact of prolonged synaptic activity on the synaptic vesicle recycling machinery, we analyzed activity-dependent uptake of the lipophilic fixable dye FM1-43FX to synaptic boutons, visualized by post-hoc immunofluorescence with antibodies against the synaptic vesicle protein SV2. Uptake of FM1-43FX applied in the presence of 50 µM PTX to induce synaptic vesicle exo- and endocytosis was slightly reduced in CHL1−/− versus CHL1+/+ neurons (Fig. 10B). Preincubation of neurons with 50 µM PTX for 30 min before application of the dye further reduced FM1-43FX uptake in CHL1−/− but not in CHL1+/+ synaptic boutons (Fig. 10B). Similar results were obtained when synaptic vesicle recycling was visualized in real time imaging using live neurons transfected with VAMP2-pHluorin, an optical probe for synaptic vesicle exo- and endocytosis: repetitive stimulation with high K+ concentrations resulted in an inhibition of the recycling response to the second stimulus in CHL1−/− but not CHL1+/+ neurons (Fig. S3). The results thus indicate that the synaptic vesicle recycling machinery is impaired under prolonged excitatory synaptic activity in CHL1−/− synapses.

## Discussion

We describe a novel role for CHL1 as a co-chaperone in the presynaptic chaperone machinery consisting of Hsc70, CSP and αSGT. The physiological significance of the interactions between CHL1 containing chaperones and proteins of the synaptic vesicle fusion machinery is underscored by our observations in CHL1−/− mice: CHL1 deficiency results in enhanced susceptibility of syntaxin 1B, SNAP25 and VAMP2 to degradation especially under stressful conditions, such as exposure to heat or prolonged synaptic activity. Syntaxin1B, SNAP25 and VAMP2 not only degrade faster in CHL1−/− neurons, but also show reduced functionality under stressful conditions manifested by decreased ability to bind to each other.

VAMP2, SNAP25, and syntaxin 1B are ‘unstructured’ proteins in a sense that they do not possess typical secondary structures such as α-helixes and β-sheets. Nevertheless, exposure to conditions close to pathological (42°C) results in a profound aggregation of these proteins without any significant changes in their overall random-coil structure. The latter observation is in agreement with the notion that protein aggregation is intrinsic to the common peptide backbone of any polypeptide chain [38]. Protein aggregation is often prevented by chaperones which bind to the aggregating proteins [39], and our data indicate that CHL1 containing chaperones have a similar function with respect to SNARE proteins both *in vitro* and *in vivo*, as indicated by increased aggregation of VAMP2 and SNAP25 in CHL1−/− brains. Accumulation of unfolded proteins may impose an increased load on the chaperone machinery in CHL1−/− mice, which is also suggested by a compensatory overexpression of chaperones in CHL1−/− brains. Over-occupancy of chaperones in CHL1−/− mice may result in their unavailability for new targets, probably accounting for a drastically lower chaperone activity towards such artificial substrate as firefly luciferase.

Recombinant Hsc70, CSP and αSGT readily bind to each other and form a trimeric complex *in vitro* [25]. We show that CHL1 splits Hsc70/CSP/αSGT complex into CHL1/CSP and CHL1/Hsc70/αSGT complexes: CHL1 competes with CSP for binding to Hsc70, which binds to the similar HPD tripeptide containing sequences within CHL1ID and the J-domain of CSP [7], [40]. On the other hand, CHL1 binds to CSP via the HPD tripeptide containing domain within CHL1ID, and this binding of CHL1 to CSP interferes with the binding of CSP to Hsc70 or αSGT. The latter observation suggests that CHL1 binds to the J-domain of CSP containing the binding site for Hsc70. In agreement, in synaptic vesicles and synaptic plasma membranes from CHL1+/+ brain tissue, Hsc70 and αSGT were found together in a complex with CHL1 but not CSP, while CSP was found in a complex with CHL1 but not Hsc70 or αSGT.

Interestingly, addition of recombinant Hsc70 to CHL1−/− synaptosomes strongly inhibited the reactivation of luciferase in these preparations. A possible explanation for the dominant-negative effect of recombinant Hsc70 is that, when added in excess, it changes the stoichiometry of the chaperone complexes in synaptosomes. In particular, it may promote dissociation of the Hsc70/CSP/αSGT trimers, which are formed in the absence of CHL1, and induce formation of αSGT/Hsc70 and CSP/Hsc70 dimers and Hsc70 monomers which have a considerably lower chaperone activity towards luciferase when compared to Hsc70/CSP/αSGT trimers [25].

Why should CHL1 disassemble the trimeric Hsc70/CSP/αSGT complex? The physiological significance of this process is underscored by analysis of chaperone activity in the presence of non-native SNAP25 and VAMP2, which activated only CHL1/Hsc70/αSGT and CHL1/CSP complexes, respectively, but not the Hsc70/CSP/αSGT complex. Importantly, ability of CSP to function independently of Hsc70 in chaperone complexes has been already shown previously [30], [32].

CHL1 may enhance the affinity of the chaperones to non-native SNAP25 and VAMP2, since recombinant CHL1ID interacts directly with non-native but not native SNAP25 and VAMP2. Because CSP and VAMP2 are localized to synaptic vesicles, binding of CHL1 to VAMP2 most probably occurs in these organelles. In agreement, the CHL1/VAMP2 complex is disrupted after synaptic vesicle exocytosis which is accompanied by redistribution of VAMP2 to the surface membrane. Furthermore, binding of CHL1 to CSP is favored by ATP, which should be more abundant in non-stimulated synaptic boutons. In contrast, SNAP25, accumulating at the synaptic surface plasma membrane, most probably binds to CHL1 at the synaptic surface membrane. In agreement, the association of CHL1 with SNAP25 is enhanced following induction of synaptic vesicle exocytosis, i.e. when SNARE complex proteins accumulate at the plasma membrane. Formation of the CHL1/Hsc70/αSGT complex that binds SNAP25 is favored by ADP, being present at higher levels in stimulated synapses. Although CHL1/Hsc70/αSGT complexes are formed at the plasma membrane, they can redistribute to synaptic vesicles following internalization of CHL1 to these organelles [7], an observation that would explain lower levels of Hsc70 and αSGT in synaptic vesicles from CHL1−/− mice.

Unlike CSP deficient mice [27], CHL1−/− mice do not exhibit gross neurodegenerative changes [2], suggesting that CHL1−/− mice compensate for abnormal function of Hsc70, CSP, and αSGT by increasing expression levels of these proteins (Fig. S2, [7]). However, in the brains of CHL1−/− mice that were maintained under normal housing conditions, SNARE complex components were found to aggregate abnormally and accumulate in lysosomes, suggesting that incorrectly folded proteins gradually accumulate in the brain. We would like to speculate that this phenomenon may relate to some of the molecular abnormalities seen in slowly developing disorders of the brain, such as schizophrenia, which is not associated with gross neurological changes as well. The etiology of schizophrenia, a disease linked to mutations in the CHL1 gene in humans [16], [17], not only includes a genetic predisposition but also an environmental underpinning [41], [42]. Changes in schizophrenic patients progress slowly until they reach a critical threshold that is manifested by the disease symptoms. Our observations are in line with this hypothesis: small, genetically determined abnormalities in synaptic transmission and synaptic protein turnover can aggravate, especially following stressful conditions, and, depending on the degree of aggravation, lead to irreversible changes in the properties of information processing in the brains of humans and mice carrying mutations in CHL1, thereby contributing to the development of the disease. Independent of this hypothesis, our observations show that the adhesion molecule CHL1 is a novel regulator of the presynaptic chaperone machinery and thus a decisive player in synaptic vesicle exocytosis in central nervous system synapses.

## Materials and Methods

### Ethics Statement

All experiments were conducted in accordance with the German and European Community laws on protection of experimental animals, and all procedures used were approved by the responsible committee of The State of Hamburg (permit ORG210).

### Antibodies

The monoclonal antibody 2C2 reacting with the cytoplasmic domains of L1 and CHL1 [2], a kind gift of Marty Grumet (Rutgers University, Piscataway, NJ, USA), and polyclonal antibodies against the extracellular domain of CHL1 and L1 [43] were as described. Goat polyclonal antibodies against Hsc70 and synaptophysin were purchased from Santa Cruz Biotechnology (Santa Cruz, California, USA). We also used rabbit polyclonal antibodies against synaptophysin (a kind gift of Reinhard Jahn, Max-Planck-Institute for Biophysics, Göttingen, Germany). Monoclonal antibody (α6F) against Na,K-ATPase and mouse monoclonal antibody against SV2 were from the Developmental Studies Hybridoma Bank (University of Iowa, USA), mouse monoclonal antibodies against γ-adaptin, SNAP25 and CSP were from BD Biosciences (San Jose, CA, USA), rat monoclonal antibodies against Hsc70 were from Stressgen (Victoria, BC, Canada), rabbit polyclonal antibodies against actin were from Sigma (St. Louis, MO, USA), chicken polyclonal antibodies against αSGT, rat monoclonal antibodies against Hsc70 and rabbit polyclonal antibodies to Lamp 2b were from Abcam (Cambridge, UK), rabbit polyclonal antibodies against caspase-3 recognizing full-length non-active and cleaved active enzyme were from Cell Signaling (Beverly, MA, USA), rabbit polyclonal antibodies against syntaxin1B and mouse monoclonal antibodies against SNAP25, and VAMP2 were from Synaptic Systems (Göttingen, Germany), mouse monoclonal antibodies against GST were from Novagen (Darmstadt, Germany), rabbit polyclonal antibodies against neuronal class III β tubulin were from Covance (Berkeley, CA, USA). Rabbit polyclonal antibodies against CSP, αSGT, and βSGT were a kind gift from Guido Meyer (Max-Planck-Institute for Experimental Medicine, Göttingen, Germany). Secondary antibodies against rabbit, goat, chick and mouse immunoglobulins (Ig) coupled to horse radish peroxidase (HRP), Cy2, Cy3 or Cy5 were from Dianova (Hamburg, Germany).

### DNA constructs

The construct encoding His_6_-tagged Hsc70 was a kind gift of Christine Knuehl (Charité, Berlin, Germany). Constructs encoding His_6_-tagged αSGT and GST-tagged Hsc70, CSP, αSGT and βSGT were generous gifts of Guido Meyer and Nils Brose (Max-Planck-Institute for Experimental Medicine, Göttingen, Germany). Constructs encoding His_6_-tagged SNAP25 and VAMP2 were kindly provided by Dirk Fasshauer (Max-Planck-Institute for Biophysical Chemistry, Göttingen, Germany). His_6_-tagged L1ID was donated by Melanie Richter (Pasquale lab, The Burnham Institute, La Jolla, USA). His_6_-tagged CHL1ID was as described [7]. Synapto-pHluorin construct was a kind gift of Gero Miesenböck (Yale University School of Medicine, New Haven, CT, USA). Constructs encoding His_6_-tagged synaptophysin and GST were from RZPD (Berlin, Germany) and Amersham Pharmacia Biotech (Freiburg, Germany), respectively.

### Recombinant proteins

Recombinant proteins were produced in *E. coli*, bound to Ni-NTA agarose beads (QIAGEN, Hilden, Germany) via the His_6_ tag or glutathione-agarose beads (Sigma) via the GST tag and used for further experiments or purified according to the manufacturer's instructions. We also used SNAP25 from ProSpec (Rehovot, Israel) and synaptophysin from Abnova (Taipei, Taiwan). CHL1-Fc was as described [1]. Human Fc was from Dianova.

### Preparation of homogenates and synaptosomes

Mouse brain homogenates were prepared in HOMO buffer (1 mM MgCl_2_, 1 mM CaCl_2_, 1 mM NaHCO_3_, 5 mM Tris, pH 7.4) containing 0.32 M sucrose (HOMO-A) and used for synaptosome isolation as described [44]. All steps were performed at 4°C. Briefly, homogenates were centrifuged at 1400 g for 10 min. The supernatant and pellet were resuspended in HOMO-A buffer and centrifuged for 10 min at 700 g. The resulting supernatants were combined and centrifuged at 17500 g for 15 min. The pellet was resuspended in HOMO-A buffer and applied on the top of a step gradient with interfaces of 0.65 M, 0.85 M, 1 M, 1.2 M sucrose in HOMO buffer. The 700 g pellets were combined, adjusted to 1 M sucrose in HOMO buffer and layered on 1.2 M sucrose in HOMO buffer. HOMO-A buffer was applied on the top of the gradient. The crude synaptosomal fractions were collected at the 1 M/1.2 M interface after centrifugation for 2 h at 100000 g and combined. The crude synaptosomal fraction was again adjusted to 1 M sucrose and layered on the top of the 1.2 M sucrose. HOMO-A buffer was applied on the top of the gradient. After centrifugation for 2 h at 100000 g, synaptosomes were collected at the 1 M/1.2 M interface, resuspended in HOMO-A buffer and pulled down by centrifugation for 30 min at 100000 g.

### Isolation and purification of synaptic vesicles

Synaptic vesicles were isolated essentially as described [45], [46]. Briefly, 4 to 5 adult mouse brains were homogenized in 320 mM sucrose/4 mM HEPES, pH 7.4, and centrifuged at 1100 g for 10 min. All steps were performed at 4°C. The resulting supernatant and pellet, which was resuspended in cold 320 mM sucrose/4 mM HEPES, pH 7.4, were centrifuged for 15 min at 9200 g and 10500 g, respectively. The resulting pellets (crude synaptosomal fraction) were lysed by diluting them in 9 volumes of ice-cold H_2_O and treated by immediate adjustment by 1 M HEPES, pH 7.4, to a final concentration of 7.5 mM HEPES. After incubation on ice for 30 min, the lysate was centrifuged at 25500 g for 20 min. The supernatant was then centrifuged for 2 h at 130000 g. The resulting pellet representing the crude synaptic vesicle fraction was resuspended in 30 mM sucrose/4 mM HEPES, pH 7.4, homogenized by passage through a 25-gauge needle, loaded on a continuous gradient of 50-800 mM sucrose/4 mM HEPES, pH 7.4, and centrifuged at 100000 g for 5h. A broad band in the 200–400 mM sucrose region containing synaptic vesicles was collected and pooled by centrifugation at 250000 g for 2h. The pellet was resuspended in 30 mM sucrose/4 mM HEPES, pH 7.4 and was found to be highly enriched in the synaptic vesicle marker synaptophysin and negative for plasma membrane marker Na,K-ATPase and endosomal marker EEA1 (not shown).

### Luciferase refolding assay

For the luciferase refolding assay, brain homogenates, synaptosomes and synaptic vesicles (with a total protein concentration 0.6, 0.23 and 0.64 mg/ml, respectively) or recombinant His_6_-SGT, GST-CSP, His_6_-Hsc70 or His_6_-CHL1ID (500 nM of each protein) were used. Before analysis, brain homogenates were lysed in 1x Luciferase cell culture lysis reagent (Promega, Madison, WI, USA) for 15 min on ice and centrifuged at 14000 g for 5 min to collect the supernatants to be used in the assay. Crude synaptosomal fraction was lysed for 30 min on ice by dilution with 9 volumes of ice-cold H_2_O, and adjusted by 1M HEPES, pH 7.4, to a final concentration of 7.5 mM HEPES. Recombinant luciferase (20 nM, QuantiLum Recombinant Luciferase, Promega) was inactivated by incubation for 2 h at 25°C in 8 M guanidine hydrochloride/2 mM DTT/30 mM Tris, pH 7.2. Inactivated luciferase was then diluted 50-fold in 50 mM KCl/30 mM HEPES, pH 7.4/2 mM DTT/3 mM MgCl_2_/1 mM ATP and mixed (1∶1) with the samples. The mixture was incubated for 1h at 30°C and the activity of the reactivated luciferase was measured using the Promega luciferase assay system and a TD-20/20 luminometer (Turner Biosystems, Sunnyvale, CA, USA). The activities of equal amounts of inactivated and native luciferase in phosphate buffered saline (PBS, pH 7.4) were set to 0% and 100%, respectively.

### ATPase assay

ATPase activity was measured by estimating inorganic phosphate released during ATP hydrolysis. Recombinant SNAP25, synaptophysin and VAMP2 were incubated for 10 min at 42°C in 25 mM HEPES buffer, pH 7.5, containing 150 mM KCl, 2 mM DTT, and 5 mM MgCl_2_. Various combinations of recombinant GST-tagged CSP and His_6_-tagged αSGT, Hsc70 and CHL1ID (3 µM of each protein) were incubated with heat-treated non-native or native SNAP25, synaptophysin or VAMP2 (0.24 µM for each protein) in 20 mM HEPES buffer, pH 7.5, containing 40 mM KCl, 1 mM (NH_4_)_2_SO_4_, 2 mM MgCl_2_, 500 µM DTT, 1.2 mM ATP for 1 h at 30°C. Inorganic phosphate released from ATP was measured using the Malachite green phosphate detection kit (R&D Systems, Wiesbaden-Nordenstadt, Germany).

### Pull down assay

In a typical pull down experiment, beads with the bound “prey” protein were incubated with the purified potential binding partners or control proteins overnight at 4°C in 3% BSA, 0.l% Tween 20, 0.1 mM phenylmethansulfonylfluoride (PMSF) and 1 mM ADP or ATP in PBS, pH 7.5. After washing with PBS containing 1% BSA, 0.1 mM PMSF, 1 mM ADP (or ATP), 0.1% Tween 20, pH 7.5, protein complexes were eluted from the beads by incubation in Laemmli buffer and analyzed by SDS-PAGE and Western blot using appropriate antibodies.

### Gel electrophoresis and immunoblotting

Proteins were separated by 8–16% SDS-PAGE and electroblotted onto 0.2 µm nitrocellulose transfer membrane PROTRAN (Schleicher & Schuell, Dassel, Germany) overnight at 40 V. To analyze protein complexes, samples containing synaptic vesicles and plasma membranes were lysed for 1 h at room temperature in 50 mM Tris-HCl buffer, pH 7.4, containing 1% Triton X-100, 1 mM Na_2_P_2_O_7_, 1 mM NaF, 2 mM NaVO_4_, 0.1 mM PMSF and complete protease inhibitor cocktail (Roche Diagnostics). After removal of Triton X-100 from the samples using Bio-Beads SM-2 (Bio-Rad, Hercules, CA, USA), 2-mercaptoethanol was added to the samples to increase protein solubility and prevent protein aggregation. Proteins were then separated by 6% PAGE and electroblotted onto 0.2 µm nitrocellulose transfer membrane PROTRAN (Schleicher & Schuell) overnight at 40 V. Immunoblots were incubated with appropriate primary antibodies followed by incubation with peroxidase-labeled secondary antibodies and visualized using Super Signal West Pico reagents (Pierce Chemical Co., Rockford, IL, USA) on BIOMAX film (Sigma). Molecular weight markers were prestained protein standards (Bio-Rad) and apoferritin (Sigma). Blots were quantified using TINA 2.09 software (University of Manchester, UK).

### Isolation of lysosomes

Lysosomes were isolated on a discontinuous OptiPrep gradient using the lysosome isolation kit (Sigma). Fractions were collected at the interfaces of the OptiPrep gradient and assayed for the activity of acid phosphatase, a hydrolase highly accumulated in lysosomes, using the acid phosphatase assay kit (Sigma). The fraction containing the highest acid phosphatase activity (at the interface between 12% and 16% of the OptiPrep) was taken as purified lysosomes. Western blot analysis confirmed that this lysosomal fraction was highly enriched in lysosomal protein Lamp2b and negative for trans-Golgi marker protein γ-adaptin.

### Isolation of protein aggregates

Protein aggregates were isolated by differential centrifugation by modifying a described protocol [47]. Adult mouse brains were homogenized in extraction buffer (60 mM HEPES, pH 7.4, 0.1% Triton X-100, 100 mM NaCl, 0.5 mM PMSF, 0.1 mM DTT and complete protease inhibitor cocktail (Roche Diagnostics)) and centrifuged at 680 g for 10 min. Resulting supernatants were centrifuged at 100000 g for 1 h. Pellets were resuspended in extraction buffer, sonificated and centrifuged at 17000 g for 30 min. Resulting pellets were resuspended in extraction buffer and centrifuged at 5000 g for 30 min. Collected pellets containing protein aggregates were analyzed by Western blot analysis. All steps were performed at 4°C.

### Co-immunoprecipitation

Homogenates from adult mouse brains were prepared in 50 mM Tris-HCl buffer, pH 7.5, containing 0.32 M sucrose, 1 mM of CaCl_2_, 1 mM MgCl_2_, and 1 mM NaHCO_3_. Samples containing 1 mg of total protein were lysed for 40 min at 4°C with lysis buffer, pH 7.5, containing 50 mM Tris-HCl, 150 mM NaCl, 1% Nonidet P-40, 1 mM Na_2_P_2_O_7_, 1 mM NaF, 1 mM EDTA, 2 mM Na_3_VO_4_, 0.1 mM PMSF and complete protease inhibitor cocktail (Roche Diagnostics). To analyze co-immunoprecipitation of CSP and VAMP2, the lysis buffer was supplemented with 1% SDS. After lysis, SDS was removed using Detergent-OUT SDS removal kit (Merck, Darmstadt, Germany). Samples were centrifuged for 5 min at 14000g at 4°C. Supernatants were cleared with protein A/G-agarose beads (Santa Cruz Biotechnology) (3 h at 4°C) and incubated with corresponding antibodies or non-specific control IgG (overnight, 4°C), followed by precipitation with protein A/G-agarose beads (1 h, 4°C). The beads were washed 3 times with lysis buffer, 2 times with TBS and boiled in Laemmli buffer. Eluted material was used for Western blot analysis.

### Circular dichroism (CD) spectroscopy

CD spectra were recorded at 20°C using a Jasco J-715 spectropolarimeter (Jasco, Gross-Umstadt, Germany) equipped with a temperature-regulated sample chamber. A 1 mm optical path length quartz cell was used to obtain spectra in the far UV region (190 to 260 nm) at a protein concentration of 0.63 µM for SNAP25 and 1 µM for VAMP2 in 10 mM MgSO_4_/4 mM DTT/50 mM HEPES, pH 7.5. The CD spectra were acquired at a scan speed of 20 nm/min and a step resolution of 0.1 nm. For each experiment, 20 individual scans were averaged, and background spectra were subtracted.

### Dynamic light scattering (DLS) measurements

Dynamic light scattering measurements were made using the DIMINIGON-A DLS device (Dierks und Partner, Hamburg, Germany) with a He-Ne laser providing radiation with a wavelength of 658 nm and an output power in the range of 10–50 mW. The samples (40 µl) were placed in a quartz cuvette and measured at a constant temperature of 20°C using an autopilot function accumulating 22 measurements per sample at every 20 s, with a wait time of 1 s.

### Enzyme-linked immunosorbent assay (ELISA)

CHL1ID (1.9 µM) was immobilized on the plastic surface of MaxiSorp 96 well plates (Nunc, Roskilde, Denmark) overnight at +4°C in PBS. Wells were then washed 3 times with PBS containing 0.05% Tween 20, blocked with 1% BSA in PBS for 1 h at room temperature, and incubated overnight at 4°C with SNAP25 (4 µM) and VAMP2 (6 µM), in PBS containing 0.05% Tween 20 and 0.1% BSA. SNAP25 and VAMP2 were either non-treated or pre-treated by exposure to 42°C as described in *ATPase assay* section. Wells were washed 3 times with PBS containing 0.05% Tween 20. Bound proteins were detected with antibodies against SNAP25 and VAMP2 followed by HRP-conjugated secondary antibodies. Protein binding was visualized by detecting HRP with the OPD reagent (Pierce, Rockford, IL, USA) that resulted in a colored product. The amount of colored product was quantified using an ELISA reader at 406 nm.

### Cultures of hippocampal neurons

Cultures of hippocampal neurons were prepared from 1- to 3-day-old mice. Neurons were grown for 14–21 days in Neurobasal A medium (Invitrogen, Carlsbad, CA, USA) supplemented with 2% B-27 (Invitrogen), glutamine (Invitrogen) and FGF-2 (2 ng/ml, R&D Systems) on glass coverslips (for live imaging and immunocytochemsitry) or in 6-well plastic plates (for biochemistry) coated with poly-D-lysine (100 µg/ml). For live cell imaging, neurons were transfected 12 days after plating using Lipofectamine 2000 (Invitrogen) according to the manufacturer's instructions.

### Immunofluorescence labeling

Immunolabeling was performed essentially as described [48]. All steps were performed at room temperature. Neurons were fixed for 15 min in 4% formaldehyde in PBS, pH 7.4, permeabilized with 0.25% Triton X-100 in PBS, pH 7.4 for 5 min and blocked with 1% BSA in PBS for 20 min. Primary antibodies were applied in 1% BSA in PBS for 2 hours and detected with corresponding secondary antibodies applied for 45 min. Coverslips were embedded in Aqua-Poly/Mount (Polysciences Inc., Warrington, PA, USA). Images were acquired at room temperature using a confocal laser scanning microscope LSM510.

### Loading of FM1-43FX in synaptic boutons

Synaptic boutons of cultured hippocampal neurons [49] were loaded with FM1-43FX by incubating neurons for 10 min in the medium containing FM1-43FX (15 µM) (Invitrogen) in the presence of 50 µM PTX (Tocris, Ballwin, MO, USA).

### Immunofluorescence and FM uptake quantification

In fixed and permeabilized neurons, labeling with SV2 antibodies was used to identify synaptic boutons that were defined as SV2 accumulations with a mean intensity of at least 30% higher than background. The background was defined as the mean intensity of pixels in the square 30x30 pixel area located in the vicinity of the synaptic bouton in the part of the image devoid of synaptic boutons. Synaptic boutons were outlined using a threshold function of the ImageJ software (National Institute of Health, Bethesda, MD, USA). Within the outlines, mean intensities of the SV2 labeling and FM dye were measured and expressed in arbitrary units defined as 8 bit pixel values of the gray scale image. In each experiment, images were acquired using identical settings and the same threshold was used for all groups.

### VAMP2-pHluorin fluorescence quantification

During recordings, live hippocampal neurons transfected in vitro with VAMP2-pHluorin were maintained at room temperature in modified Tyrode solution (150 mM NaCl, 4 mM KCl, 2 mM MgCl_2_, 10 mM glucose, 10 mM HEPES and 2 mM CaCl_2_ (pH 7.4, ∼310 mOsm)) or 47 mM K+ buffer (modified Tyrode solution containing equimolar substitution of KCl for NaCl) to induce synaptic vesicle exo- and endocytosis [50]. All procedures were performed in the presence of 10 µM CNQX and 50 µM AP-5 to prevent recurrent activation elicited by AMPA and NMDA receptors, respectively. Changes in VAMP2-pHluorin fluorescence were monitored with time by acquiring images using a laser scanning microscope LSM510 (Zeiss, Jena, Germany) with 1 s intervals. With these recorded time series, synaptic boutons were identified as accumulations of VAMP2-pHluorin fluorescence that appeared following the first application of 47 mM K+ and were at least 30% brighter in intensity than the background VAMP2-pHluorin fluorescence before application of 47 mM K+. VAMP2-pHluorin clusters were outlined using ImageJ when VAMP2-pHluorin fluorescence reached the maximum following the first application of 47 mM K+. These outlines were then used to measure VAMP2-pHluorin fluorescence intensity on all images that were recorded before, during and after the first and second application of 47 mM K+.

### Statistical analysis

Statistical significance of the differences observed by Western blot analysis and by luciferase reactivation and ATPase assays was analyzed using the paired t-test or repeated measures ANOVA Dunnett's multiple comparison test as indicated in the figure legends. Differences in FM1-43 uptake were compared by the t-test. Numbers of observations and compared groups are indicated in Figure legends.

## Supporting Information

Figure S1 Hsc70 and CSP, but not alphaSGT and CHL1 possess intrinsic ATPase and protein refolding activities. A - ATPase activity estimated by measuring free inorganic phosphate released from ATP in the presence of recombinant Hsc70, CSP, alphaSGT, or CHL1ID and heat-treated recombinant luciferase. Graph shows mean ± SEM (n = 6). Note, that Hsc70 and CSP but not CHL1ID and alphaSGT possess detectable intrinsic ATPase activity. *,+p<0.05, paired t-test (when compared to the group incubated with Hsc70 (*), or with CSP (+)). B - Graph shows mean levels of activity ± SEM (n = 6) of the luciferase, that was reactivated in the presence of recombinant Hsc70, CSP, alphaSGT, or CHL1ID. Values were normalized to the luciferase reactivation levels in the presence of Hsc70. Note, that luciferase reactivation in the presence of CHL1ID and alphaSGT was lower than in the presence of Hsc70 and CSP. Taking into account that CHL1ID and alphaSGT do not show detectable ATPase activity, luciferase reactivation in the presence of these two proteins most probably occurs via spontaneous refolding of luciferase. *,+p<0.05, paired t-test (when compared to the group incubated with Hsc70 (*), or with CSP (+)).
(0.06 MB TIF)

Figure S2 Levels of alphaSGT are reduced in CHL1−/− synaptosomes. CHL1+/+ and CHL1−/− brain homogenates and synaptosomes isolated from CHL1+/+ and CHL1−/− brains were probed by Western blot with antibodies against alphaSGT and betaSGT. Representative blots are shown. Graphs show mean optical densities of the blots ± SEM (n = 6) with optical density for CHL1+/+ probes set to 100%. Note that levels of alphaSGT, but not betaSGT are increased in CHL1−/− versus CHL1+/+ brain homogenates and decreased in CHL1−/− versus CHL1+/+ synaptosomes. *p<0.05, paired t-test.
(0.21 MB TIF)

Figure S3 Repetitive stimulation inhibits synaptic vesicle recycling in CHL1−/− cultured hippocampal neurons. To directly analyze the impact of CHL1 deficiency on synaptic vesicle recycling, we transfected cultured hippocampal neurons with a pH-sensitive green-fluorescent protein fused to the lumenal domain of VAMP2 (VAMP2-pHluorin), providing an optical probe to follow synaptic vesicle exo- and endocytosis in real time [53]: Synaptic vesicle exocytosis exposes pHluorin to the fluorescence-permissive culture medium, while pHluorin endocytosis to synaptic vesicles results in quenching of fluorescence in the acidified environment of the vesicle lumen. Neurons were challenged twice with 47 mM K+ for 90 s with a recovery in 4 mM K+ following each application of 47 mM K+. Diagrams (left) show mean levels ± SEM of synapto-pHluorin fluorescence intensities in synaptic boutons as a function of time. The values were normalized to the synapto-pHluorin fluorescence intensity before stimulation. Black bars indicate time of 47 mM K+ application. Time lapse recordings of axons of the transfected neurons showed that in CHL1+/+ neurons both applications of 47 mM K+ resulted in similar increases in VAMP2-pHluorin fluorescence in synaptic boutons, indicating that comparable numbers of synaptic vesicles were exocytosed. Substitution of 47 mM K+ with 4 mM K+ resulted in a gradual decline in VAMP2-pHluorin fluorescence intensity, indicating synaptic vesicle endocytosis. In contrast to CHL1+/+ neurons, CHL1−/− neurons responded with a pronounced increase in VAMP2-pHluorin fluorescence levels in synaptic boutons only to the first application of 47 mM K+, while the second response was strongly reduced. Graph (right) shows mean ratio ± SEM of the amplitudes (Amp) of synapto-pHluorin fluorescence intensity increase in response to the first and second application of 47 mM K+. * p<0.05, t-test (n = 10 synapses from five CHL1+/+ neurons and n = 20 synapses from six CHL1−/− neurons were analyzed). A reduction in the second response could be due to the impaired endocytosis of synaptic vesicles in CHL1−/− synaptic boutons following the first stimulus that would result in unavailability of synaptic vesicles for exocytosis during the second stimulus. Indeed, the endocytosis of VAMP2-pHluorin following the first application of 47 mM K+ was slower and more incomplete in CHL1−/− versus CHL1+/+ synaptic boutons in agreement with our previous report [7]. However, 81 +/− 5.2% of VAMP2-pHluorin fluorescence was quenched in CHL1−/− synaptic boutons by the end of the recovery time after the first stimulus indicating that impaired endocytosis in CHL1−/− synaptic boutons may account for only 20% reduction in the response to the second stimulus. In fact, the second response was reduced by 81 +/− 17% when compared to the first one. Furthermore, the changes in responses defined as the ratio of the amplitudes of VAMP2-pHluorin fluorescence increase after the first and second application of 47 mM K+ did not correlate with the efficiency of VAMP2-pHluorin endocytosis following the first stimulus (correlation coefficient = −0.083) indicating that the impairment in synaptic vesicle exocytosis in response to the second stimulus cannot be explained only by impaired synaptic vesicle endocytosis after the first stimulus. Interestingly, the amplitude of the first response was slightly higher in CHL1−/− versus CHL1+/+ neurons, possibly due to increased levels of CSP in CHL1−/− synaptic boutons [54]. This observation is in agreement with the previously reported enhanced basal synaptic transmission in CHL1−/− mice [9].
(0.10 MB TIF)

Table S1 Changes in ATPase activity in different combinations of CHL1ID, Hsc70, CSP and alphaSGT in the presence of non-native versus native SNAP25, VAMP2 or synaptophysin. Recombinant CHL1ID, Hsc70, CSP and alphaSGT in the indicated combinations were incubated with heat-treated non-native and native SNAP25, VAMP2 or synaptophysin. Table shows ATPase activity in the presence of non-native proteins normalized to ATPase activity in the presence of native proteins set to 100%. ATPase activity values observed in the presence of non-native proteins that are statistically different (paired t-test, n≥6) from the values obtained for native proteins are highlighted in yellow. ND - not determined.
(0.32 MB TIF)
