# Supplementary figures and images for: CHL1 Is a Selective Organizer of the Presynaptic Machinery Chaperoning the SNARE Complex
Source: PLoS One. 2010 Aug 11;5(8):e12018. doi: 10.1371/journal.pone.0012018 (PMC2920317; doi:10.1371/journal.pone.0012018)

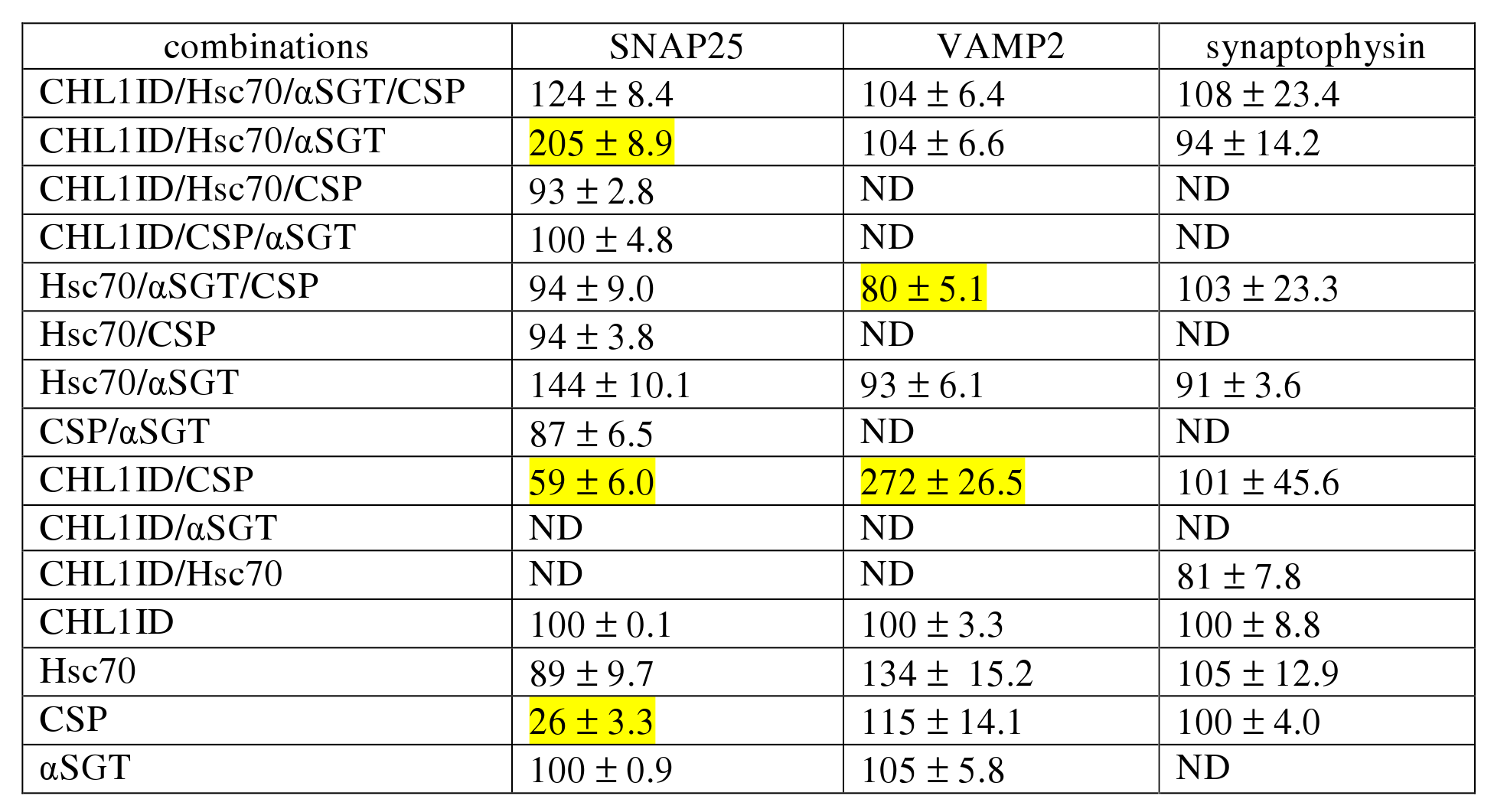

Supplement: Table S1 — Changes in ATPase activity in different combinations of CHL1ID, Hsc70, CSP and alphaSGT in the presence of non-native versus native SNAP25, VAMP2 or synaptophysin. Recombinant CHL1ID, Hsc70, CSP and alphaSGT in the indicated combinations were incubated with heat-treated non-native and native SNAP25, VAMP2 or synaptophysin. Table shows ATPase activity in the presence of non-native proteins normalized to ATPase activity in the presence of native proteins set to 100%. ATPase activity values observed in the presence of non-native proteins that are statistically different (paired t-test, n≥6) from the values obtained for native proteins are highlighted in yellow. ND - not determined. (0.32 MB TIF) [file pone.0012018.s004.tif]
